# Dismantling cognitive-behaviour therapy for panic disorder: a systematic review and component network meta-analysis

**DOI:** 10.1017/S0033291717003919

**Published:** 2018-01-25

**Authors:** Alessandro Pompoli, Toshi A. Furukawa, Orestis Efthimiou, Hissei Imai, Aran Tajika, Georgia Salanti

**Affiliations:** 1 MD, Psychiatric Rehabilitation Clinic Villa San Pietro, Trento, Italy; 2 Departments of Health Promotion and Human Behavior, Kyoto University Graduate School of Medicine/School of Public Health, Kyoto, Japan; 3 Institute of Social and Preventive Medicine (ISPM), University of Bern, Bern, Switzerland; 4 Department of Hygiene and Epidemiology, University of Ioannina School of Medicine, Ioannina, Greece

**Keywords:** Cognitive-behaviour therapy, component network meta-analysis, panic disorders

## Abstract

Cognitive-behaviour therapy (CBT) for panic disorder may consist of different combinations of several therapeutic components such as *relaxation*, *breathing retraining*, *cognitive restructuring*, *interoceptive exposure* and/or *in vivo exposure*. It is therefore important both theoretically and clinically to examine whether specific components of CBT or their combinations are superior to others in the treatment of panic disorder. Component network meta-analysis (NMA) is an extension of standard NMA that can be used to disentangle the treatment effects of different components included in composite interventions. We searched MEDLINE, EMBASE, PsycINFO and Cochrane Central, with supplementary searches of reference lists and clinical trial registries, for all randomized controlled trials comparing different CBT-based psychological therapies for panic disorder with each other or with control interventions. We applied component NMA to disentangle the treatment effects of different components included in these interventions. After reviewing 2526 references, we included 72 studies with 4064 participants. *Interoceptive exposure* and *face-to-face setting* were associated with better treatment efficacy and acceptability. *Muscle relaxation* and *virtual-reality exposure* were associated with significantly lower efficacy. Components such as *breathing retraining* and *in vivo exposure* appeared to improve treatment acceptability while having small effects on efficacy. The comparison of the most *v.* the least efficacious combination, both of which may be provided as ‘evidence-based CBT,’ yielded an odds ratio for the remission of 7.69 (95% credible interval: 1.75 to 33.33). Effective CBT packages for panic disorder would include *face-to-face* and *interoceptive exposure* components, while excluding *muscle relaxation* and *virtual-reality exposure*.

## Introduction

Panic disorder is an anxiety disorder characterized by the recurrence of unexpected panic attacks, in which an intense fear accompanied by a series of bodily and/or cognitive symptoms develops abruptly, without an apparent external cause (American Psychiatric Association, 2013). In the general population, about one-quarter of people suffering from panic disorder also have agoraphobia (Kessler *et al.* 2006). Panic disorder is common in the general population, with a life-time prevalence of 3.7% without agoraphobia and 1.1% with agoraphobia (Kessler *et al.* 2006). In primary care settings, panic syndromes have been reported to have a prevalence of around 10% (King *et al.* 2008).

Three types of intervention are recommended for treating individuals with panic disorder (NICE, 2011). The intervention for which there is evidence for the longest duration of effect is psychological therapy, followed by pharmacological therapy and self-help. Among various psychological therapies, guidelines recommend the use of cognitive-behaviour psychotherapy (CBT) (American Psychiatric Association, 1998; Royal Australian & New Zealand College of Psychiatrists, 2003; NICE, 2011; Katzman *et al.* 2014). In line with these recommendations, a recent Cochrane review and network meta-analysis (Pompoli *et al.* 2016) found CBT to be the most efficacious treatment among other competing psychological therapies.

CBT for panic disorder is usually administered according to the manuals by Clark & Salkovskis (Clark & Salkovskis, 1986), Barlow & Craske (Barlow & Craske, 2000) or their adaptations. In its classical form, CBT consists of various therapeutic components, mainly represented by psychoeducation, breathing retraining, muscle relaxation, cognitive restructuring, interoceptive exposure and *in vivo* exposure. In clinical practice and in the research literature, we find therapies which correspond to the general definition of CBT, but which consist of different sets of therapeutic components. It has been observed, however, that some combinations of these components (i.e. some specific forms of CBT) may lead to better results than their isolated administration (Sanchez-Meca *et al.* 2010). It is therefore clinically and theoretically important to understand the relative efficacy of different components of CBT for panic disorder. Such a component hierarchy will help minimize the complexity of the treatments being offered and may provide an improvement in terms of time, money and efforts for both patients and clinicians. In addition, streamlining CBT intervention would simplify therapist training and possibly allow a broader range of clinicians to be trained, thus improving overall accessibility to CBT.

Component network meta-analysis (NMA) is a newly developed meta-analysis methodology where various components of different therapies can be dismantled and compared (Welton *et al.* 2009). We have conducted a systematic review of randomized controlled trials (RCTs) comparing various CBT treatments for panic disorder with or without agoraphobia, in terms of remission, response and dropouts, and applied the component NMA in order to answer the clinical question ‘Are particular components of CBT for panic disorder or combinations of such components more effective than others?’ The objective of the present systematic review is to obtain a hierarchy of the components involved in CBT for panic disorder according to their efficacy and acceptability and to identify those components associated with most and least favourable outcomes.

## Methods

The analyses have been conducted in adherence to a pre-specified protocol registered in PROSPERO (CRD42015027601) and the results are reported following the PRISMA extension statement for network meta-analysis (Hutton *et al.* 2015).

### Study eligibility criteria

We included all RCTs that compared any form of CBT against another form of CBT or any other psychotherapeutic control intervention. We included adult patients of both sexes, with a primary diagnosis of panic disorder with or without agoraphobia. We included studies in which diagnoses were made according to operationalized diagnostic criteria. Table 1 presents the 12 different components of interest and their definition. We included a psychological therapy or a control intervention as long as it could be regarded as a combination of these 12 components. Table 2 provides a clarification of how the various forms of CBT were conceptualized according to a component-level perspective.

**Table 1.** List of included components and their definitions

Component		Description
*w*	Waiting component	Participants are aware that they will receive an active treatment after a waiting phase. This component was considered present even when non-specific psychotherapy was provided while the participants were aware that they could receive the ‘active’ intervention after the waiting period was over
*pl*	Placebo effect	Effect of an intervention due to the patients’ belief that they are receiving some form of treatment
*ps*	Psychological support	Effect of an intervention due to various non-specific techniques (e.g. encouragement, rationalizing and reframing, anticipatory guidance, etc.) administered within the context of a therapeutic alliance (Winston *et al.* 2004). Considered present even in self-help format if personal encouragement was provided to proceed with the self-help material
*pe*	Psychoeducation	It consists in providing patients information about their psychological disease
*br*	Breathing retraining	It consists in teaching patients various techniques aimed at correcting those respiratory patterns thought to elicit or sustain panic attacks
*mr*	Progressive/applied muscle relaxation	*Progressive muscle relaxation* is aimed at reducing general tension and achieving a body state that lowers the risk for stressors to provoke a panic attack (Bernstein & Borkovec, 1973). In the so-called *applied relaxation* (Ost, 1987), relaxation training and exposure are combined
*cr*	Cognitive restructuring	Psychotherapeutic process of learning to identify and modify irrational or maladaptive thoughts (such as catastrophic misinterpretation of bodily sensations) using strategies such as Socratic questioning, thought recording and guided imagery
*ine*	Interoceptive exposure	Graded exposure to bodily sensations that accompany panic
*ive*	*In vivo* exposure	Graded exposure to real-life situations perceived as threatening
*vre*	Virtual reality exposure	Graded exposure to virtual reality simulations reproducing real-life situations perceived as threatening
*3w*	Third wave components	Various techniques aimed at helping patients to develop more adaptive emotional responses to situations, such as the ability to observe symptomatic processes without overly identifying with them or without reacting to them in ways that cause further distress (Roemer *et al.* 2008)
*ftf*	Face-to-face setting	Administration of therapeutic components in a face-to-face setting (rather than through self-help means)

Group format was not considered a component because in a previous review and NMA comparing various psychological therapies for the treatment of panic disorder (Pompoli et al, 2016), we did not detect any association between the relative treatment effects and the difference of therapy delivery (individual *v.* group) format.

**Table 2.** Conceptualization of various forms of CBT according to a component-level perspective

Interventions or controls	Possible decompositions into components
Waiting list (WL)	*w* (±*pl* ± *pe* ± *ps* ± *ftf*)
No treatment (NT)	
Attention/psychological placebo (APP)	*pl* + *ftf*
Self-help psychoeducation (SH-PE)	*pl* + *pe*
Face-to-face psychoeducation (PE)	*pl* + *pe* + *ftf*
Supportive psychotherapy (SP)	*pl* (±*pe*) + *ps* + *ftf*
Self-help physiological therapy (SH-PT)	*pl* (±*pe* ± *ps*) + *br*/*mr*
Face-to-face physiological therapy (PT)	*pl* (±*pe* ± *ps*) + *br*/*mr* + *ftf*
Self-help cognitive therapy (SH-CT)	*pl* (±*pe* ± *ps* ± *br* ± *mr*) + *cr*
Face-to-face cognitive therapy (CT)	*pl* (±*pe* ± *ps* ± *br* ± *mr*) + *cr* + *ftf*
Self-help behaviour therapy (SH-BT)	*pl* (±*pe* ± *ps* ± *br* ± *mr*) + *ine*/*ive*/*vre*
Face-to-face behaviour therapy (BT)	*pl* (±*pe* ± *ps* ± *br* ± *mr*) + *ine*/*ive*/*vre* + *ftf*
Self-help cognitive-behaviour therapy (SH-CBT)	*pl* (±*pe* ± *ps* ± *br* ± *mr*) + *cr* + *ine*/*ive*/*vre*
Face-to-face cognitive-behaviour therapy (CBT)	*pl* (±*pe* ± *ps* ± *br* ± *mr*) + *cr* + *ine*/*ive*/*vre* + *ftf*
Self-help *third wave* CBT (SH-3W)	*pl* (±*pe* ± *ps* ± *br* ± *mr* ± *cr* ± *ine* ± *ive* ± *vre*) + *3w*
Face-to-face *third wave* CBT (3W)	*pl* (±*pe* ± *ps* ± *br* ± *mr* ± *cr* ± *ine* ± *ive* ± *vre*) + *3w* + *ftf*

*w*, waiting component; *pl*, placebo effect; *ftf*, face-to-face setting; *pe*, psychoeducation; *ps*, psychological support; *br*, breathing retraining; *mr*, muscle relaxation; *cr*, cognitive restructuring; *ine*, interoceptive exposure; *ive*, *in vivo* exposure; *vre*, virtual reality exposure; *3w*, third wave components.

Note that, abbreviations in uppercase (eg. WL) stand for interventions/controls, whereas abbreviations in lowercase italics stand for therapeutic components (eg. *w*). Components in parentheses are elective/optional.

Symbols: ‘+’ means ‘and’; ‘±’ means ‘with or without’; ’/’ means ‘and/or’.

Therapies could be of any duration. We included both individual and group therapies. Pharmacological co-administration was allowed as long as there were no systematic differences in drug administration between study arms.

### Outcomes

The primary outcome of the study was remission of panic disorder with or without agoraphobia in the short term (measured as close to 3 months as possible, with a maximum of 6 months). Remission was defined as a dichotomous outcome expressing the number of patients who reached a satisfactory end-state, as defined by the original investigators (e.g. achieving a score of 7 or less at end-point on Panic Disorder Severity Scale (PDSS)). Secondary outcomes included response and dropouts for any reason in the short term. The response was defined as a dichotomous outcome expressing the number of patients who had a substantial improvement from baseline, as defined by the original investigators (e.g. at least 40% reduction in PDSS scores from baseline).

### Search strategy

In March 2015, we conducted a comprehensive and systematic search of all psychological therapies for panic disorder in order to identify relevant studies for a Cochrane review that has recently been published (Pompoli *et al.* 2016). We updated and re-assessed these search results according to relevant inclusion and exclusion criteria for this study in November 2015. Online Supplement 1 provides the details of the databases searched, along with supplementary searches and search strategies used.

### Data collection

Two review authors independently screened the titles and abstracts of references identified by the electronic search and reviewed the full text of any study deemed potentially eligible. Reviewers resolved disagreements by discussion.

Two review authors used a structured manualized data collection form to independently extract the data from the included studies. Any disagreement, including the determination of constituent components of interventions, was resolved either by discussion or by consultation with a third member of the review team. We tried to contact the study authors for all relevant missing data. When this attempt failed, we calculated the number of remitted and/or responding participants according to a validated imputation method (Furukawa *et al.* 2005).

Two review authors independently assessed the risk of bias (RoB) of the included studies using the Cochrane Risk of Bias assessment tool (Higgins *et al.* 2011).

### Data synthesis

For the data synthesis, we employed a component NMA model, similar to the model described in Welton *et al.* (2009). This model is an extension of the standard NMA model, where the effect of each composite intervention is dismantled after modelling the component-specific effects. We hence differentiated between the effect of a CBT component and the effect of an intervention (combination of components). In the primary analyses we assumed additivity of component effects, i.e. the total effect of each composite intervention was assumed equal to the sum of effects of the included components. According to this model, adding a component *c* to a composite intervention *X* will lead to an increase (or decrease) of the odds of the event by an amount only dependent on *c*, and not on *X*. We denote the corresponding component-specific incremental odds ratio by *iOR*_*c*_ so that *iOR*_*c*_ = *OR*_(*X*+*c*) *v*. (*X*)_. Combining these component-specific incremental odds ratio allows the estimation of odds ratios between any two composite interventions. For example, *OR*_(*ftf*+*ps*) *v*. (*w*)_ = *iOR*_*ftf*_ × *iOR*_*ps*_/*iOR*_*w*_ (see Table 1 for the abbreviations of the components). Consequently, a large *iOR*_*c*_ suggests that component *c* has a large impact on the outcome.

We assessed the assumptions involved in the primary analyses as follows. First, the NMA requires transitivity of treatment effects across the network. We evaluated the assumption of transitivity by comparing the distribution of effect modifiers across studies grouped by treatment comparisons and components. In addition, we employed the design-by-treatment interaction test to assess the consistency of the network statistically (Higgins *et al.* 2012). Second, the additivity assumption of the component NMA was evaluated by comparing the relative intervention effects in appropriate subgroups of studies; e.g. studies *X* + *Z v. X* should, on average, estimate the same relative treatment effects as *Y* + *Z v. Y* studies. We also ran sensitivity analyses to examine interaction terms among important components.

In previous analyses, we found substantial evidence of small study effects for the comparison of active treatments *v.* waiting list with respect to their efficacy (Pompoli *et al.* 2016). Consequently, our primary model for response and remission was a component network meta-regression with the variance of the study log-odds-ratio as a covariate. No adjustment for small study effects was done for dropout rate. In order to interpret the magnitude of the estimated heterogeneity (*τ*), for each outcome we compared the estimated heterogeneity with the expected value, defined in accordance with the empirical distributions for comparing non-pharmacological interventions for a subjective outcome (Turner *et al.* 2012) (median for heterogeneity *τ* = 0.36; 95% predictive distribution range = 0.07–1.82).

We fitted the model in a Bayesian framework, using OpenBUGS (Lunn *et al.* 2009) and uncertainty in the results was conveyed by the 95% credible intervals (CrI). The model accounted for correlations induced by multi-arm studies and a half-normal prior to heterogeneity standard deviation was used. Online Supplement 2 provides the details of the statistical methods used.

## Results

### Results of the search

Our search identified 2526 references. After removal of duplicates and screening based on title and abstract, 616 references (353 studies) were retrieved for a full inspection. Finally, 72 studies, representing 4064 participants, were included in quantitative analyses (online Supplement 3).

### Description of studies

Online Supplement 4 summarizes the references and characteristics of the included and excluded studies. Table 3 shows the number of study arms that included each component. Apart from *third wave components*, which were administered only in two studies (each contributing to only one outcome), all other components were well represented in the network. The percentage of agreement among raters for the identification of components ranged from 99.0 to 99.7%.

**Table 3.** Number and percentage of study arms including each component and estimates of corresponding incremental odds ratios (iOR) parameter, with 95% Credible Intervals (CrI), for remission, response and dropouts

	Remission		Response		Dropouts	
Component	*n* (%) of included arms	iOR (95% CrI)	*n* (%) of included arms	iOR (95% CrI)	*n* (%) of included arms	iOR (95% CrI)
Third-wave components (3w)	1 (0.8%)	1.97 (0.34–14.44)	0 (0.0%)		1 (0.75%)	0.55 (0.08–3.63)
Interoceptive exposure (ine)	54 (40.3%)	1.49 (0.94– 2.36)	48 (38.7%)	1.43 (0.94–2.16)	58 (37.9%)	0.95 (0.53–1.70)
Face-to-face setting (ftf)	82 (61.2%)	1.27 (0.57– 2.66)	78 (62.9%)	1.79 (0.93–3.53)	97 (63.4%)	0.54 (0.17–1.51)
Cognitive restructuring (cr)	69 (51.2%)	1.11 (0.73– 1.73)	61 (49.2%)	0.87 (0.60–1.27)	74 (48.4%)	1.00 (0.60–1.70)
Placebo effect (pl)	101 (75.4%)	0.97 (0.35–2.48)	94 (75.8%)	1.04 (0.29–3.35)	116 (75.8%)	15.18 (1.67–347.23)
Breathing retraining (br)	45 (33.6%)	0.84 (0.54– 1.26)	40 (32.3%)	1.16 (0.81–1.67)	51 (33.3%)	0.71 (0.45–1.14)
Psychoeducation (pe)	89 (66.4%)	0.84 (0.45– 1.52)	84 (67.7%)	1.17 (0.68–2.03)	100 (65.4%)	0.93 (0.43–2.03)
Psychological support (ps)	90 (67.2%)	0.79 (0.34– 1.90)	84 (67.7%)	0.86 (0.40–1.84)	105 (68.6%)	1.38 (0.44–4.66)
*In vivo* exposure (ive)	64 (47.8%)	0.78 (0.50– 1.22)	61 (49.2%)	0.96 (0.66–1.46)	71 (46.4%)	0.86 (0.49–1.48)
Virtual-reality exposure (vre)	7 (5.22%)	0.71 (0.25– 1.99)	8 (6.5%)	0.41 (0.19–0.84)	6 (3.9%)	1.77 (0.58–5.37)
Muscle relaxation (mr)	30 (22.4%)	0.59 (0.40– 0.90)	29 (23.4%)	0.64 (0.46–0.88)	35 (22.9%)	1.07 (0.64–1.77)
Waiting component (w)	34 (25.4%)	0.38 (0.12– 1.23)	30 (24.9%)	0.80 (0.21– 2.86)	38 (24.8%)	5.26 (0.59–121.51)
*τ*		0.40 (0.05– 0.73)		0.23 (0.02–0.55)		0.53 (0.11–0.91)
Coefficient of the variance in the meta-regression		−1.96 (−2.72 to −1.30)		−1.66 (−2.26 to −1.08)		−

Larger values of the parameters indicate respectively, larger remission, response and dropout rates. Components are ordered according to iORs for remission, from the most to the least beneficial.

The lower part of the table reports the median heterogeneity standard deviation (*τ*) for the three outcomes and the regression coefficient for the two outcomes for which analyses were adjusted for small study effects.

The risk of bias of many included studies was judged as unclear or high in various important domains. Online Supplement 5 provides the RoB assessment for each domain and for each study. The percentage of agreement among raters for the assessment of RoB ranged from 75 to 96%.

### Assessments of transitivity, inconsistency and additivity assumption

We compiled a table of important trial and patient characteristics (therapy duration, percentages of agoraphobic, depressed and drug-treated patients) across treatment comparisons and components. Its visual inspection showed that those effect modifiers were likely to be similarly distributed across the networks of treatments and components, although there was missing information from several studies (online Supplement 2). Overall there was little concern about the plausibility of the transitivity assumption.

After exploring data for inconsistencies, first at the treatment level and then at the component level, we found no strong evidence of inconsistency for any of the explored outcomes but the power of this analysis is expected to be low (online Supplement 6).

We did not find any strong evidence against the additivity assumption (online Supplement 6). Sensitivity analyses examining several clinically relevant interaction terms in the model (*ftf* with *ine*, *ftf* with *cr*, *pe* with *ine*, *cr* with *ive*, *br* with *ine*, *br* with *ive*) did not alter the results: none of these interactions was statistically significant (online Supplement 7).

#### Primary outcome: remission of panic disorder

**Fig. 1.**
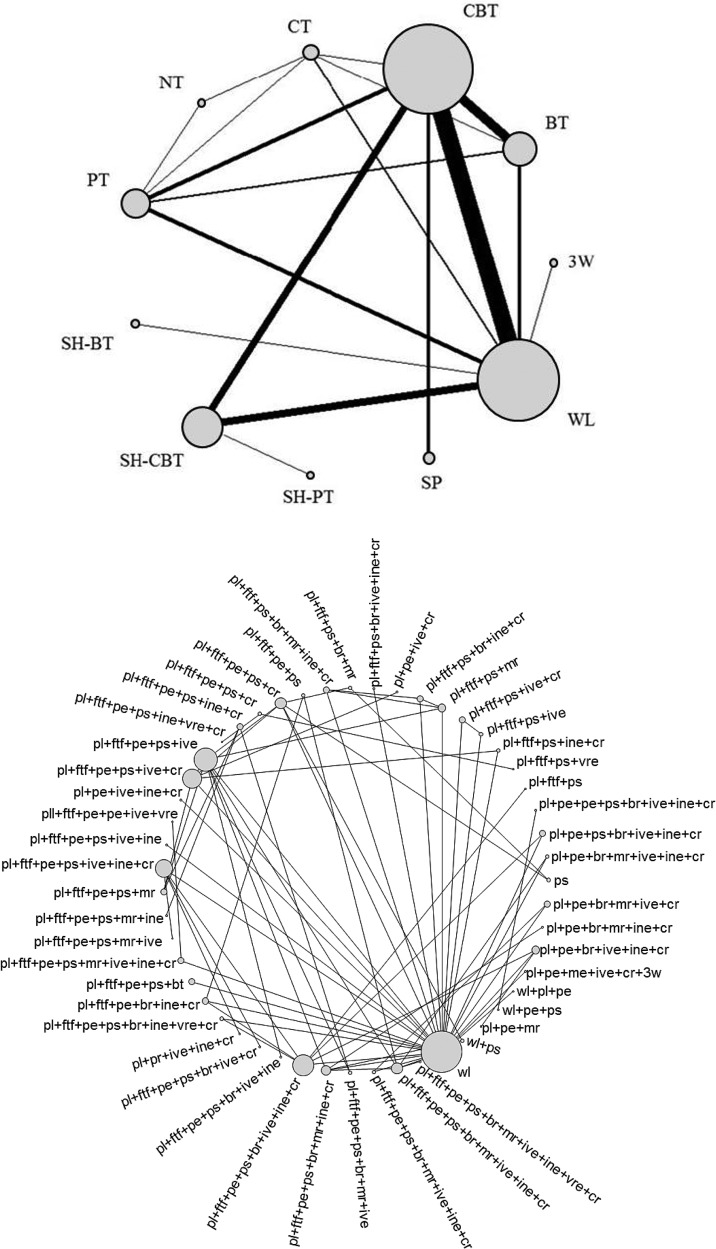
The network structure for short-term remission at the composite-interventions level (top) and at the component level (bottom).

Data regarding remission were available from 60 studies, with a total of 134 study arms (3556 patients). Figure 1 shows the network at the treatment level and at the component level.

Including the variance of the treatment effect in the meta-regression model reduced the median heterogeneity standard deviation from *τ* = 0.85 (0.56–1.22) to *τ* = 0.40 (0.05–0.73) and the regression coefficient was estimated to be −1.96 (−2.72 to −1.30) providing strong evidence for the presence of small study effects. Heterogeneity was found to be close to the expected values (Turner *et al.* 2012). The results from the network meta-regression are presented in Table 3.

The inclusion of *third-wave components*, *interoceptive exposure*, *face-to-face setting* and *cognitive restructuring* in a composite intervention apparently led to higher odds of remission but uncertainty was large, as seen from the very wide CIs for the corresponding iOR (Table 3). Conversely, *placebo effect*, *breathing retraining*, *psychoeducation*, *psychological support*, *in-vivo exposure* and *virtual reality exposure* were found to decrease the odds of remission, but again with large uncertainty in the estimates. However, including *muscle relaxation* in a complex treatment was shown to significantly reduce the efficacy in terms of short-term remission. Results were favourable for *third-wave components*, but with very large uncertainty. As expected, the *waiting component* (which is mainly administered without other components as a supposedly inert control condition) showed the smallest mean *iOR*.

Based on the findings of Table 3 (and excluding *third wave components*, which was only informed by a single study, and for which the estimate was very imprecise) we can identify the CBT associated with the largest remission rates as consisting of (*ftf* + *pl* + *pe* + *ps* + *cr* + *ine*) and the least effective CBT as (*pl* + *pe* + *ps* + *br* + *mr* + *ive* + *vre*); the components of *pl*, *pe* and *ps* are included here as would be expected in typical CBT programs but in the calculation of relative efficacy their effects are cancelled out and the best treatment would provide an average relative increase in the odds of remission by almost 700% (OR 7.69 CrI 1.75 to 33.33).

### Response to the interventions

A total of 55 studies (124 study arms including 3275 patients) provided evidence on the response. There was evidence of small study effects in the comparisons of active interventions *v.* waiting list. Heterogeneity was found to be within the range of expected values (Turner *et al.* 2012). Table 3 gives the values of the *iOR* values for response estimated from the component network meta-regression. Taken together, results about response were in general agreement with those for remission. Results were more precise for *muscle relaxation* and *virtual reality exposure*, which appeared to reduce the odds of response.

### Dropout rate from the study

Data regarding dropouts was available from 68 studies, with a total of 153 study arms (3705 patients). Table 3 gives the estimates of the *iOR* parameters for dropouts, where values smaller than 1 are associated with higher treatment acceptability. Heterogeneity was again found to be within the range of expected values (Turner *et al.* 2012).

The low estimate for *iOR*_*ftf*_ suggests that dropouts were generally less frequent in face-to-face therapies than in self-help therapies. Components such as *breathing retraining* and *in vivo exposure* seemed to marginally improve treatment acceptability; the inclusion of *psychoeducation*, *interoceptive exposure*, *cognitive restructuring* and *muscle relaxation* showed negligible influence on dropouts. Finally, *psychological support* and *virtual reality exposure* were associated with lower acceptability, although the corresponding estimate came with a lot of uncertainty. The *placebo effect* was associated with the highest and the *waiting component* with the second highest odds of dropout. Because all active interventions are administered together with the *placebo effect*, the estimate for this component can be seen as a sort of an average acceptability of the active treatments. Therefore, our results suggest that the risk of dropout was on average higher in active treatments as a whole than in waiting list (people placed on the waiting list were more likely to return for the assessment at end of a trial than people in active treatments). Results were again favourable but very imprecise for *third-wave components*.

## Discussion

The results of this component NMA seem to support our original hypothesis that, among the wide range of therapeutic components that fall within CBT, there are important differences in terms of efficacy and acceptability. As a consequence, the selection of different combinations of components can greatly influence the outcome of CBT for panic disorder, explaining at least part of the heterogeneity observed in previous studies examining this psychological therapy (Sanchez-Meca *et al.* 2010; Pompoli *et al.* 2016).

Based on the analyses for remission after selecting the specific combination of components in order to have the ‘most efficacious CBT’ and the ‘least efficacious CBT’, we would include *cognitive restructuring* (*cr*) and *interoceptive exposure* (*ine*) in the former, and *breathing retraining* (*br*), *muscle relaxation* (*mr*), *in vivo exposure* (*ive*) and *virtual reality exposure* (*vre*) in the latter. In the context of typical CBT, characterized by simultaneous presence of *pl*, *pe* and *ps*, the comparison of such two CBT versions, that is (*ftf* + *pl* + *pe* + *ps* + *cr* + *ine*) *v.* (*pl* + *pe* + *ps* + *br* + *mr* + *ive* + *vre*), would yield an OR of 7.69 (CrI 1.75 to 33.33) in favour of the ‘most efficacious’ CBT. On the other hand, if we compare the above defined ‘most efficacious CBT’ and what could be a commonly delivered CBT, comprising for example (*ftf* + *pl* + *pe* + *ps* + *cr* + *br* + *ive*), we would still have an OR of 2.33 (CrI 1.01 to 5.26). These contrasts are not only statistically significant but also have strong clinical implications.

In our previous review (Pompoli *et al.* 2016) we found that, in terms of short-term remission, CBT appeared to be significantly superior to behaviour therapy, with an OR of 1.79 (CrI 1.02 to 3.13), which suggested that the co-administration of cognitive and behavioural therapeutic components was superior to the administration of behavioural components alone. Another review (Sanchez-Meca *et al.* 2010) had previously concluded that the combination of exposure, relaxation/breathing techniques and cognitive therapy may represent the most effective treatment for panic disorder, with smaller effect sizes for any of these components if administered alone.

The present study, however, goes one step further to suggest which particular components, within the broad class of cognitive or behavioural approaches to treat panic disorder, are likely to be more beneficial. First, among behavioural components, *interoceptive exposure* (*ine*) tends to be associated with a better outcome than *in vivo exposure* (*ive*). Similar findings were reported in previous studies, which showed the positive effects of adding *ine* to *ive* alone (Ito *et al.* 1996; Craske *et al.* 1997), while showing less benefits in adding *ive* to *ine* alone (de Ruiter *et al.* 1989; Craske *et al.* 2003).

It must be noted that, compared with *interoceptive exposure* (*ine*), the inclusion of *cognitive restructuring* (*cr*) was shown to influence positive outcomes to a lesser degree. The administration of other components such as *breathing retraining* (*br*), *psychoeducation* (*pe*) and *placebo effect* (*pl*) was also shown to have a small influence on treatment outcome in terms of both response and remission. Interestingly, *breathing retraining* (*br*) is among those components which showed a low impact on positive outcomes, although it appeared to improve treatment acceptability. These findings are coherent with previous studies questioning the utility of *breathing retraining* (*br*) in addition to other CBT components (Schmidt *et al.* 2000) or comparing *breathing retraining* (*br*) to *interoceptive exposure* (*ine*) (Craske *et al.* 1997).

On the other hand, the inclusion of *muscle relaxation* (*mr*) was shown to be related to a significantly worse outcome both in terms of remission and response, although this component did not appear to influence treatment acceptability. This finding is coherent with a previous review which found that CBT was superior to relaxation therapy in all panic-related domains, although having no differences in terms of drop-out rates (Siev & Chambless, 2007).

Although two previous reviews, exploring self-help *v.* face-to-face CBT for various anxiety disorders (Cuijpers *et al.* 2010; Lewis *et al.* 2012), did not detect important differences between the two delivery forms when analyses were restricted to studies focusing on panic disorder, a more recent Cochrane review (Olthuis *et al.* 2015) found evidence in favor of face-to-face CBT, compared with therapist-guided internet CBT, in terms of anxiety reduction (three studies: SMD 0.29; 95% CI 0.03–0.54). Our study also suggests that the better format for the administration of CBT is the *face-to-face setting*, which leads to a better outcome both in terms of response and remission. Furthermore, it also seems to improve patients’ adherence to the treatment, reducing the number of dropouts. Finally, we found only two studies exploring *third-wave components* (Karekla, 2004; Berger *et al.* 2014), only one of which contributed data. Further studies exploring third-wave CBTs for panic disorder are warranted.

We were unable to detect any interaction among the components when we added interaction terms as would be expected from clinical or theoretical notions (e.g. Would the efficacy of interoceptive exposure or cognitive restructuring be stronger in face-to-face rather than otherwise? Would psychoeducation facilitate interoceptive exposure? Would cognitive restructuring enhance the efficacy of *in vivo* exposure? Could breathing retraining be used as a counterproductive safety behaviour during interoceptive or *in vivo* exposures?). However, it should be noted that our data were sparse and generally had low power to detect interactions. Thus, our findings should not be interpreted as ruling out the possibility of synergistic/antagonistic effects between some CBT components. Future large studies may still be able to detect clinically important interactions.

This review has some weaknesses. First of all, the high number of included studies together with the complexity of statistical analyses took us a long time to complete the review since the search date. Second, despite our efforts, we have probably been unable to include all unpublished studies, as suggested by the evidence of SSE affecting both remission and response. Third, it must be noted that some results came with a high degree of uncertainty as regards each individual component, possibly due to the limited number of relevant studies and heterogeneity among them. In the previous NMA comparing various psychological therapies for the treatment of panic disorder (Pompoli *et al.* 2016), however, we did not detect any association between the relative treatment effects and study-level characteristics such as year of publication, percentage of drug-treated patients, percentage of patients with comorbid depression, percentage of agoraphobic patients and therapy delivery setting (individual *v.* group therapy). Fourth, included studies were often assessed as being at unclear risk of bias for random sequence generation, allocation concealment or selective outcome reporting and a high percentage of studies were assessed as being at high risk of outcome assessment bias, attrition bias and researcher allegiance bias. In addition, different studies used variable definitions of remission or response. While we expect that such differences would more strongly affect absolute measures of efficacy (What percentage of patients respond?) and less strongly relative measures of efficacy (How much more likely are patients to respond on treatment than on control?) (Furukawa *et al.* 2002), this variability may be one reason that has contributed to uncertainty of estimates in the present analyses. Fifth, the definitions and identifications of components in the broad range of CBT are unavoidably arbitrary to some degree. However, we tried to be as transparent as possible by providing explicit definitions and we were able to achieve satisfactory agreement in the judgments between two independent reviewers. Finally, we did not include any long-term outcome, aware of the limited availability of relevant data (Pompoli *et al.* 2016).

This study has some important strengths, however. To our knowledge, this study is among the first attempts to implement a component-level network meta-analyses within the context of a comprehensive and methodologically rigorous systematic review, and represents a pioneering approach to disentangle the individual contributions of every single component pertaining to a complex intervention such as CBT for panic disorder. The high number of included studies, the lack of evidence against the assumptions of transitivity or additivity in the networks and the coherence between results for remission and response, suggest the robustness of our findings.

The identification of important differences between components of CBT has strong implications for clinical practice by contributing to a much more precise identification of those therapeutic components that should be included (or excluded) when offering CBT to patients affected by the panic disorder.

More effective CBT packages for panic disorder would include *face-to-face* and *interoceptive exposure* components, while the inclusion of *muscle relaxation* or *virtual reality exposure* would decrease the overall efficacy of such CBT packages. The inclusion of other components, such as *psychoeducation*, *cognitive restructuring*, *breathing retraining* and *exposure in vivo*, seems to have a lower impact on treatment outcome, and could be appropriate depending on theoretical and pragmatic considerations.

Although some components are associated with worse treatment outcome, one must keep in mind that, both in terms of remission and response, all components appear to be beneficial when compared with inert waiting list (the only exception is *virtual reality exposure* for response). This means that such components cannot be considered ‘detrimental’; rather, it can be hypothesized that, given that a treatment takes a limited amount of time, administering less effective components may lead to a worse/limited administration of the more effective ones, finally leading to a less positive clinical result.

The results from this study also have important implications for future research in complex interventions. Researchers should evaluate the possibility to exploit the used methodology in order to reach a better understanding of advantages and disadvantages of various components included in complex interventions, such as psychotherapies and many other public health interventions.

## Supplementary material

For supplementary material accompanying this paper visit http://dx.doi.org/10.1017/S0033291717003919. click here to view supplementary material
