# Supplementary material for: Dismantling cognitive-behaviour therapy for panic disorder: a systematic review and component network meta-analysis
Source: Psychol Med. 2018 Jan 25;48(12):1945–53. doi: 10.1017/S0033291717003919 (PMC6137372; doi:10.1017/S0033291717003919)
Supplement: Supplementary file 1 [file S0033291717003919sup001.docx]

**Supplementary materials**

- **Supplement 1: Details of the search strategy**
- **Supplement 2: Component network meta-analysis (with OpenBUGS codes)**
- **Supplement 3: Prisma flow diagram of study selection process**
- **Supplement 4: References and Characteristics of included, excluded and other studies**
  - **eTable 1. Characteristics of included studies**
  - **eTable 2. Characteristics of ongoing studies**
  - **eTable 3. Characteristics of studies awaiting classification**
  - **eTable 4. Characteristics of excluded studies**
- **Supplement 5: Details of Risk of Bias (RoB) assessments for each domain and for each study**
- **Supplement 6: Assessments of inconsistency and additivity assumption for the three outcomes**
- **Supplement 7: Sensitivity analyses examing six clinically suspected interactions**

**Supplement 1: Details of the search strategy**

The main searches (March 2015) were conducted in the Cochrane Depression, Anxiety and Neurosis group (CCDAN) specialised register, which collates weekly updated searches of MEDLINE (1950-), EMBASE (1974-) and PsycINFO (1967-); quarterly searches of the Cochrane Central Register of Controlled Trials (CENTRAL) and review specific searches of additional databases. Reports of trials were sourced from international trials registers such as World Health Organization’s trials portal (ICTRP), drug companies, handsearching of key journals, conference proceedings and other (non-Cochrane) systematic reviews and meta-analyses.

Supplementary searches were conducted on PubMed and on trial registries. Further supplementary searches included: the check of the reference lists of all included studies and relevant systematic reviews; the personal query to trialists and subject experts for the individuation of unpublished studies; the check of the citation index Web of Science; the search of the grey literature database OpenSIGLE. No language restrictions were applied in any search.

The updated search was conducted on the Cochrane Central Register of Controlled Trials (CENTRAL) and on PubMed after protocol registration, using search strategies similar to those described for the main searches.

**CCDANCTR-References Register Search**

1. (therap* or psychotherap*) [ti,ab]

2. Psychotherapy [kw]

3. (acceptance* or commitment* or “activity scheduling” or adlerian or art or aversion or brief or “client cent*” or cognitive* or color or colour or compassion-focused or “compassion* focus*” or compassionate or conjoint or conversion or conversational or couples or dance or dialectic* or diffusion or distraction or eclectic or (emotion and focus*) or emotion-focus* or existential or experiential or exposure or expressive or family or focus-oriented or “focus oriented” or freudian or gestalt or “group” or humanistic or implosive or insight or integrative or interpersonal or jungian or kleinian or logo or marital or metacognitive or meta-cognitive or milieu or morita or multimodal or multi-modal or music or narrative or nondirective or non-directive or “non directive” or nonspecific or non-specific or “non specific” or “object relations” or “personal construct” or “person cent*” or person-cent* or persuasion or play or ((pleasant or pleasing) and event*) or primal or problem-focused or “problem focused” or problem-solving or “problem solving” or process- experiential or “process experiential” or psychodynamic or “rational emotive” or reality or “reciprocal inhibition” or relationship* or reminiscence or restructuring or rogerian or schema* or self-control* or “self control*” or “short term” or short-term or sex or “social effectiveness” or “social skill*” or socio-environment* or “socio environment*” or “solution focused” or solution-focused or “stress management” or supportive or time-limited or “time limited” or “third wave” or transference or transtheoretical or validation)

4. (abreaction or “acting out” or “age regression” or ((assertive* or attention or autogenic or mind or sensitivity) and train*) or autosuggestion or “balint group” or ((behavior* or behaviour*) and (activation or therap* or treatment or contracting or modification)) or bibliotherap* or biofeedback or catharsis or *cognitive* or *CBT* or “mind training” or counsel* or “contingency management” or countertransference or “covert sensitization” or “eye movement desensiti*” or EMDR or “crisis intervention” or “dream analysis” or “emotional freedom” or “free association” or “functional analys*” or griefwork or hypno* or imagery or meditation* or “mental healing” or mindfulness* or “panic program” or psychoanaly* or psychodrama or psychoeducat* or (psycho* and support*) or psychotherap* or relaxation or “role play*” or “self analysis” or “self esteem” or “self-help or “self help” or “sensitivity training” or “support group*” or therapist or “therapeutic technique*” or “transactional analysis”)

5. ((1 or 2) and 3) or 4

6. panic

7. (5 and 6)

**PubMed search strategy**

(((”randomized controlled trial“[Publication Type]) OR (”controlled clinical trial“[Publication Type]) OR (”clinical trials as topic“[MeSH Terms]) OR ((randomized[Title/Abstract]) OR randomised[Title/Abstract]) OR (randomly[Title/Abstract]) OR (placebo[Title/Abstract]) OR (trial[Title])) NOT (”animals“[MeSH Terms] NOT ”humans“[MeSH Terms])) AND ((”psychother- apy“[MeSH Terms]) OR (psychotherap* OR psychoanaly* OR psychodynamic OR psychodrama OR psychoeducat*[Title/Abstract])) AND ((”agoraphobia“[MeSH Terms]) OR (”panic disorder“[MeSH Terms]) OR (”panic“[MeSH]) OR (panic OR agoraphobi*[Title/ Abstract]))

**Supplement 2. Component network meta-analysis**

If we assume a two-arm study $i$ comparing intervention X (comprising, for example, components $pl$ and $pe$) versus intervention Y (comprising, for example, components $pl$and $ftf$), then, using the log-odds ratios as an effect measure, the random effects, component NMA model is written as follows:

|  | $logit\left( p_{i,j} \right)=\left\{ \begin{aligned} \mu_{i} for intervention X \\ \mu_{i}+\delta_{i} for intervention Y \end{aligned} \right.$  $\delta_{i}\sim Normal\left( d_{Y}-d_{X},\tau^{2} \right)$, | (1) |
| --- | --- | --- |

where $p_{i, j}$ denotes the probability of having an event in treatment arm $j$ of study $i$ and $\tau$ the heterogeneity standard deviation, assumed to be common for all log-odds ratios in the network. In order to disentangle the effect of each individual component we employed the additivity assumption, i.e. the effect of a composite treatment is the sum of effects of its components. In the running example this implies that $d_{X}=d_{pl}+d_{pe}$ and $d_{Y}=d_{pl}+d_{ftf}.$ See Welton et al.^1^ for additional details on the model. The target parameters of the model are $d_{c},$ where $c= \{w, pl, pe, ps, br, mr,cr, ine, ive,vre,3w,ftf\}$. These correspond to the log-transformation of the ${iOR}_{c}$ parameters mentioned in the main paper. In order to account for the existence of SSE we adjust the model by regressing the treatment effects vs. their variance, only for studies comparing active treatments vs. waiting list. I.e. we assume that $\delta_{i}\sim Normal\left( d_{Y}-d_{X}+\beta\times var_{i}\times I_{i},\tau^{2} \right)$, where $I_{i}$ is a dummy variable equal to 1 if study $i$ compares an active treatment to waiting list, 0 otherwise. The parameter $\beta$ quantifies the SSE. For the case of studies comparing more than two treatments (multi-arm studies), we use multivariate distributions that take into account the correlations induced. We assumed equal heterogeneity variances for all comparisons in the network^2^.

We fitted our model using OpenBUGS^3^. We used uninformative prior distributions for the treatment effects and for all coefficients in the meta-regression analyses we performed, i.e. $N (0,1000)$. A minimally informative prior was used for the heterogeneity parameter, i.e. $N\left( 0,1 \right)I(0,)$. For all analyses we ran two chains of 40,000 samples, and we discarded the first 10,000 samples. We evaluated convergence of the model by inspecting the posterior distributions and by using the Brooks-Gelman-Rubin diagnostic. As a sensitivity analysis we repeated the analyses with a different prior distribution for heterogeneity,$U(0.5)$; we found little differences in results.

***Assessment of the transitivity assumption***

NMA models generally rest on the assumption of transitivity, i.e. effect modifiers have a similar distribution across treatment comparisons in the network^4,5^. In order to assess the plausibility of this assumption we summarized important trial- and patient-level characteristics for each pairwise comparison for which direct evidence was available in the network. We then visually inspected the similarity of factors that were considered to be effect modifiers (duration of the therapy, percentages of agoraphobic, depressed and drug-treated patients). We also investigated the inclusion and exclusion criteria of all trials in the network to make sure that patients, treatments and outcomes in the trials were sufficiently similar in all aspects that could modify the treatment effect.

Intransitivity in the network may manifest as inconsistency in the data. Inconsistency refers to the statistical disagreement between direct and indirect evidence in the network^4,5^. In order to evaluate inconsistency we employed two approaches: the loop-specific approach^6^ and the design-by-treatment interaction model^7,8^. We assessed inconsistency of the network at the component-level network (Figure 3). To improve power, we also assessed inconsistency at the composite treatment-level, i.e. for the ‘grouped’ network where each treatment (as in Table 1) defined a different node (Figure 3). For both networks, we report the number of inconsistent loops (for the loop-specific approach), and the global test for inconsistency for the design-by-treatment interaction model.

We fitted the loop-specific approach using the ifplot command in Stata^9^. We fitted the design-by-treatment inconsistency model in R, using the package netmeta^10^.

***Assessment of the additive treatment effects assumption***

The component-level NMA model we described assumes that the effect of each composite treatment is the sum of the effects of its components. Additivity implies that a study comparing $X$ vs $Y$ estimates the same relative effect with a study that compares $X+Z$ vs $Y+Z$, where $X, Y$ and $Z$can be any component $\{w, pl, pe, ps, br, mr, cr, ine, ive, vre,3w, ftf\}$, or combination therof. It follows that additivity also implies that studies comparing $X+Z$ vs $X$ estimate the same relative effects as studies $Y+Z$ vs $Y$. In order to check this assumption, we identified groups of studies with different designs that estimate the same relative effect and checked their corresponding estimates. Important differences would shed doubts on the validity of this assumption. We did not have enough data to explore more advanced models that explore a synergistic or antagonistic association between components^1^.

***Subgroup analysis and investigation of heterogeneity***

In a previous review and NMA comparing various psychological therapies for the treatment of panic disorder^11^, we did not detect any association between the relative treatment effects and study-level characteristics such as year of publication, percentage of drug-treated patients, percentage of patients with comorbid depression, percentage of agoraphobic patients and therapy delivery setting (individual versus group therapy). This might have been due to low power, and we did not expect that power would be considerably increased in this updated component-level network. Therefore, we did not plan any subgroup analysis.

***Investigation of transitivity assumption***

In order to check the plausibility of transivitity assumption we compiled a table of important intervention and patients’ characteristics (below) and visually inspected it in order to check whether potential effect modifiers were similarly distributed across comparisons. There was overall little concern about the plausibility of the transitivity assumption.

| **STUDY INFORMATION** | | **INTERVENTION** | **PATIENTS’ CHARACTERISTICS** | | |
| --- | --- | --- | --- | --- | --- |
| **Comparison** | **Study ID** | **Duration (weeks/modules)** | **Agoraphobia (%)** | **Drug therapy (%)** | **Depression (%)** |
| 3W vs CBT | Karekla 2004 | 10 | NA | NA | NA |
| APP vs CBT | Beck 1987 | 8 to 12 | NA | NA | NA |
| APP vs CT | Tyrer 1988 | 6 | NA | 0 | 0 |
| APP vs PE | Rees 1999 | 3 | 83 | NA | 11 |
| BT vs BT | De Beurs 1995 | 12 | 100 | NA | NA |
| BT vs BT | Ito 1996 | 10 | 100 | NA | 34 |
| BT vs BT | Michelson 1985 | 12 | 100 | 0 | 42 |
| BT vs BT vs PT | Al Kubaisy 1992 | 6 | 100 | NA | NA |
| BT vs BT vs WL | Gloster 2010 | NA | 100 | 0 | 43 |
| BT vs CBT | Burke 1997 | 10 | 100 | NA | NA |
| BT vs CBT | Hoffart 1995 | 6 | 100 | 0 | NA |
| BT vs CBT | Malbos 2011 | 10 | 100 | NA | NA |
| BT vs CBT | Meuret 2010 | 16 | 100 | NA | NA |
| BT vs CBT | Salkovskis 1999 | 1 | 100 | NA | 42 |
| BT vs CBT vs CBT | De Ruiter 1989 | 8 | 100 | NA | NA |
| BT vs CBT vs CT vs WL | Williams 1996 | 8 | 92 | 63 | 52 |
| BT vs CBT vs PT | Ost 1993 | 12 | 100 | NA | NA |
| BT vs CBT vs WL | Clark 1994 | 12 to 24 | NA | NA | NA |
| BT vs CBT vs WL | Ost 2004 | 14 to 16 | 100 | 52 | 12 |
| BT vs CBT vs WL | Cottraux 2009 | 12 | 100 | 0 | 0 |
| CBT vs CBT | Choi 2003 | 12 | 100 | NA | 0 |
| CBT vs CBT | Craske 1997 | 12 | 100 | NA | NA |
| CBT vs CBT | Craske 2003 | 16 | 82 | 38 | NA |
| CBT vs CBT | Perez-Ara 2010 | NA | 100 | NA | NA |
| CBT vs CBT vs CBT vs WL | Dow 2000 | 6 to 12 | 76 | NA | NA |
| CBT vs CBT vs PT vs WL | Barlow 1989 | 15 | NA | NA | NA |
| CBT vs CBT vs SH-CBT | Power 2000 | 12 | NA | NA | NA |
| CBT vs CBT vs WL | Botella 2004 | 9 | 83 | 67 | 27 |
| CBT vs CBT vs WL | Clark 1999 | 12 to 24 | 85 | 32 | 29 |
| CBT vs CBT vs WL | Meyerbroker 2011 | NA | 100 | NA | NA |
| CBT vs CBT vs WL | Schmidt 1997a | 12 | 58 | 59 | 17 |
| CBT vs CBT vs WL | Schmidt 2000 | 12 | NA | 54 | 20 |
| CBT vs CBT vs WL | Sharp 2004 | 12 | NA | NA | NA |
| CBT vs PT | Korrelboom 2013 | 7 | 79 | NA | NA |
| CBT vs PT | Ost 1995 | 12 | 21 | NA | NA |
| CBT vs SH-CBT | Carlbring 2004 | 10 | 51 | 31 | 6 |
| CBT vs SH-CBT | Kiropoulos 2006 | 6 to 12 | 58 | 48 | 10 |
| CBT vs SH-CBT vs WL | Gould 1993 | 4 to 7 | NA | NA | NA |
| CBT vs SH-CBT vs WL | Lidren 1994 | 8 | 83 | 39 | 0 |
| CBT vs SP | Addis 2004 | 14 | 73 | 65 | 39 |
| CBT vs SP | Craske 1995 | 4 | 67 | 0 | NA |
| CBt vs SP | Shear 1994 | 15 | 92 | 0 | 19 |
| CBT vs WL | Carter 2003 | 11 | 100 | 0 | NA |
| CBT vs WL | Craske 2005a | 10 | NA | 0 | 36 |
| CBT vs WL | Hendriks 2010 | 14 | 100 | 53 | NA |
| CBT vs WL | Klosko 1988 | 15 | NA | NA | NA |
| CBT vs WL | Meulenbeek 2008 | 8 to 12 | NA | NA | NA |
| CBT vs WL | Reinecke 2013 | 1 to 4 | 93 | 0 | 7 |
| CBT vs WL | Schmidt 1997b | 12 | NA | NA | 21 |
| CBT vs WL | Scott 1995 | 7 | NA | NA | NA |
| CBT vs WL | Telch 1993 | 8 | NA | 61 | 34 |
| CT vs CT vs WL | Muncy 1991 | 6 | NA | 0 | 63 |
| CT vs NT vs PT | Beck 1994 | 10 | NA | 0 | 23 |
| CT vs WL | Beck 1992 | 8 to 12 | 18 | 52 | 35 |
| PT vs CBT | Milrod 2015 | 16 | 78 | 34 | 23 |
| PT vs PT vs WL | Griegel 1995 | 2 to 4 | NA | NA | NA |
| PT vs PT vs WL | Wollburg 2011 | 5 to 8 | NA | NA | 9 |
| PT vs WL | Goldstein 2000 | 4 | 100 | 46 | 0 |
| PT vs WL | Meuret 2008 | 4 | NA | NA | NA |
| SH-3W vs SH-3W vs WL | Berger 2014 | 8 | NA | NA | NA |
| SH-BT vs WL | Woolaway-Bickel 2008 | 5 | 82 | 36 | 9 |
| SH-CBT vs CBT | Bergstrom 2010 | 10 | NA | NA | NA |
| SH-CBT vs SH-CBT vs WL | Richards 2006 | 8 | 78 | 36 | 9 |
| SH-CBT vs SH-PT | Carlbring 2003 | 28 | 91 | 50 | 13 |
| SH-CBT vs WL | Bell 2012 | 12 | NA | NA | NA |
| SH-CBT vs WL | Carlbring 2001 | 10 | NA | 64 | 7 |
| SH-CBT vs WL | Carlbring 2006 | 10 | NA | 54 | 33 |
| SH-CBT vs WL | Gould 1995 | 4 to 14 | NA | NA | NA |
| SH-CBT vs WL | Nordin 2010 | 10 | 60 | 38 | 68 |
| SH-CBT vs WL | Wims 2010 | 8 | NA | 31 | NA |
| SH-CBT vsCBT | Hecker 2004 | 10 | 85 | 63 | NA |
| WL (plus *pe*) vs WL | Helbig2009 | 8 | 100 | NA | NA |

NA: Not available

| **Component** | **Duration of therapy in weeks (SD)** | **% agoraphobia (SD)** | **% drug-treated patients (SD)** | **% depressed patients (SD)** |
| --- | --- | --- | --- | --- |
| *wl* | 10 (3) | 80 (28) | 37 (25) | 16 (18) |
| *pl* | 11 (5) | 86 (22) | 31 (26) | 16 (16) |
| *ftf* | 10 (5) | 89 (18) | 27 (27) | 17 (18) |
| *pe* | 11 (5) | 86 (21) | 30 (25) | 16 (17) |
| *ps* | 10 (5) | 87 (20) | 28 (26) | 17 (17) |
| *br* | 10 (4) | 83 (15) | 35 (24) | 14 (13) |
| *mr* | 11 (5) | 89 (17) | 21 (25) | 5 (9) |
| *ive* | 11 (5) | 89 (19) | 33 (24) | 16 (16) |
| *ine* | 11 (5) | 82 (22) | 34 (24) | 16 (14) |
| *vre* | 10 (3) | 98 (6) | 22 (38) | 0 (0) |
| *cr* | 11 (5) | 84 (19) | 34 (24) | 13 (16) |
| *3w* | 10 (2) | NA | NA | NA |
| **overall** | 10 (5) | 85 (22) | 33 (25) | 16 (17) |

NA: Not available

***Cited references***

1. Welton NJ, Caldwell DM, Adamopoulos E, Vedhara K. Mixed Treatment Comparison Meta-Analysis of Complex Interventions: Psychological Interventions in Coronary Heart Disease. American Journal of Epidemiology 2009; 169(9):1158–1165.
2. Higgins JPT, Whitehead A. Borrowing Strength from External Trials in a Meta-Analysis. Statistics in Medicine 1996;15:2733–49
3. Lunn D, Spiegelhalter D, Thomas A, Best N. The BUGS project: Evolution, critique and future directions. Statistics in Medicine 2009;28:3049–3067.
4. Salanti G. Indirect and mixed-treatment comparison, network, or multiple-treatments meta-analysis: many names, many benefits, many concerns for the next generation evidence synthesis tool. Research Synthesis Methods 2012;3(2):80–97.
5. Efthimiou O, Debray TPA, van Valkenhoef G et al. on behalf of GetReal Methods Review Group. GetReal in network meta-analysis: a review of the methodology. *Research Synthesis Methods* 2016,7(3):236-263.
6. Bucher HC, Guyatt GH, Griffith LE, Walter SD. The results of direct and indirect treatment comparisons in meta-analysis of randomized controlled trials. Journal of Clinical Epidemiology 1997;50(6):683-91.
7. Higgins JPT, Jackson D, Barrett JK, Lu G, Ades AE, White IR. Consistency and inconsistency in network meta- analysis: concepts and models for multi-arm studies. Research Synthesis Methods 2012;3(2):98–110.
8. White IR, Barrett JK, Jacksona D, Higgins JPT. Consistency and inconsistency in network meta-analysis: model estimation using multivariate meta-regression. Research Synthesis Methods 2012;3:111–25.
9. Chaimani A, Higgins JPT, Mavridis D, Spyridonos P, Salanti G. Graphical Tools for Network Meta-Analysis in STATA. PloS One 2013, 8:e76654. doi: 10.1371/journal.pone.0076654
10. Gerta Rücker, Guido Schwarzer, Ulrike Krahn and Jochem König (2016). Netmeta: Network Meta-Analysis using Frequentist Methods. R package version 0.9-1. https://CRAN.R-project.org/package=netmeta
11. Pompoli A, Furukawa TA, Imai H, Tajika A, Efthimiou O, Salanti G. Psychological therapies for panic disorder with or without agoraphobia in adults: a network meta-analysis. Cochrane Database of Systematic Reviews 2016.

***OpenBUGS codes for fitting the component NMA model (accounting for SSE)***

**Data required:**

ns: number of studies

nt: number of components

ref: reference component (arbitrarily chosen)

na: vector(with ns elements),with the number of treatment arms per RCT

n: data structure (dimensions=ns*max(na)), number of patients per treatment arm

r: data structure (dimensions=ns*max(na)), number of events per treatment arm

wl, ftf, pl, etc.: data structure (dimensions=ns*max(na)), with elements 1 or 0, serving as indicators of whether a component was included in a treatment arm. E.g. if wl[5,2]=1 then in study 5, arm 2 included a wl component

model {

for(i in 1:ns) {

w[i,1]<- 0

theta[i,1]<- 0

##binomial likelihood of number of events for each arm k of study i

for (k in 1:na[i]) {r[i,k] ~ dbin(p[i,k],n[i,k])}

##parameterization of the 'true' effect of each comparison

##of arm k vs. baseline arm (1) of study i

logit(p[i,1])<- u[i]

for (k in 2:na[i]) {

logit(p[i,k])<- u[i] + theta1[i,k]

theta1[i,k]<-theta[i,k]+Beta*(var[i,k])*I[i,k]

##distribution of random effects

theta[i,k] ~ dnorm(md[i,k],precd[i,k])

## to be used for SSE adjustment

lamda.1[i,k]<- equals(r[i,k],0)

lamda.2[i,k]<- equals(n[i,k],r[i,k])

lamda.3[i,k]<- equals(r[i,1],0)

lamda.4[i,k]<- equals(n[i,1],r[i,1])

lamda.a[i,k]<- max(lamda.1[i,k],lamda.2[i,k])

lamda.b[i,k]<- max(lamda.3[i,k],lamda.4[i,k])

lamda[i,k]<-max(lamda.a[i,k],lamda.b[i,k])

var[i,k]<-1/(r[i,k]+(0.5*lamda[i,k]))+1/(r[i,1]+(0.5*lamda[i,k]))

+1/(n[i,k]-r[i,k]+(0.5*lamda[i,k]))+1/(n[i,1]-r[i,1]+(0.5*lamda[i,k]))

## This is an indicator of whether there was a comparison vs. WL (to be used for SSE adjustment)

I[i,k]<-equals(wl[i,k],1)-equals(wl[i,1],1)

## accounting for the correlation between effect sizes estimated in multi-arm trials

md[i,k]<- mean[i,k] + sw[i,k]

w[i,k]<- (theta[i,k] - mean[i,k])

sw[i,k]<- sum(w[i,1:k-1])/(k-1)

precd[i,k]<- prec *2*(k-1)/k

##consistency equations

mean[i,k] <-A[i,k]-B[i,k]

A[i,k]<-d[1]*(1-equals(wl[i,k],0)) + d[3]*(1-equals(ftf[i,k],0)) + d[4]*(1-equals(pe[i,k],0)) + d[5]*(1-equals(ps[i,k],0)) + d[6]*(1-equals(br[i,k],0)) + d[7]*(1-equals(mr[i,k],0))+ d[8]*(1-equals(ive[i,k],0)) + d[9]*(1-equals(ine[i,k],0)) + d[10]*(1-equals(vre[i,k],0))+ d[11]*(1-equals(cr[i,k],0)) + d[2]*(1-equals(w3[i,k],0))+ d[12]*(1-equals(pl[i,k],0))

B[i,k]<-d[1]*(1-equals(wl[i,1],0))+ d[3]*(1-equals(ftf[i,1],0)) + d[4]*(1-equals(pe[i,1],0)) + d[5]*(1-equals(ps[i,1],0)) + d[6]*(1-equals(br[i,1],0)) + d[7]*(1-equals(mr[i,1],0))+ d[8]*(1-equals(ive[i,1],0)) + d[9]*(1-equals(ine[i,1],0)) + d[10]*(1-equals(vre[i,1],0))+ d[11]*(1-equals(cr[i,1],0)) + d[2]*(1-equals(w3[i,1],0))+ d[12]*(1-equals(pl[i,1],0))

}}

Beta ~ dnorm(0,0.01)

##prior distribution for log-odds in baseline arm of study i

for (i in 1:ns) {u[i] ~ dnorm(0,.01)}

##prior distribution for heterogeneity

tau ~ dnorm(0,1)I(0,)

prec<- 1/pow(tau,2)

tau.sq<- pow(tau,2)

##prior distribution for basic parameters

for(k in 1:nt) {d[k] ~ dnorm(0,.001)}

##OR for each comparison

for(i in 1:(nt-1)) {

for (j in (i+1):nt) {

OR[j,i]<- exp(d[j] - d[i])

LOR[j,i]<- d[j] - d[i]}}

for(j in 1:(ref-1)){ORref[j]<- exp(d[j] - d[ref])}

for(j in (ref+1):nt) {ORref[j]<- exp(d[j] - d[ref])}

#Ranking of components#

for(k in 1:nt) {

order[k]<-nt+1 -rank(d[],k)

# this is when the outcome is negative - change to 'order[k]<- t+1-rank(d[],k) when the outcome is negative

most.effective[k]<-equals(order[k],1)

for(j in 1:nt) {effectiveness[k,j]<- equals(order[k],j)

cumeffectiveness[k,j]<- sum(effectiveness[k,1:j])}}

##SUCRAS

for(k in 1:nt) {

SUCRA[k]<- sum(cumeffectiveness[k,1:(nt-1)]) /(nt-1)

}

##Fit of the Model

for(i in 1:ns) {

for (k in 1:na[i]) {

Darm[i,k]<- -2*( r[i,k] *log(n[i,k]*p[i,k]/ r[i,k])+(n[i,k] - r[i,k])*log((n[i,k]-n[i,k]* p[i,k])/(n[i,k]- r[i,k]))) }

D[i]<- sum(Darm[i,1:na[i]]) }

D.bar<- sum(D[])}

**Supplement 3: Prisma flow diagram of study selection process**

**Supplement 4: References and Characteristics of included, excluded and other studies**

**References of included studies**

**Addis 2004 {published data only}**

Addis ME, Hatgis C, Cardemil E, Jacob K, Krasnow AD, Mansfield A. Effectiveness of cognitive-behavioral treatment for panic disorder versus treatment as usual in a managed care setting: 2-year follow-up. Journal of Consulting and Clinical Psychology 2006;**74**(2):377–85.

*Addis ME, Hatgis C, Krasnow AD, Jacob K, Bourne L, Mansfield A. Effectiveness of cognitive-behavioral treatment for panic disorder versus treatment as usual in a managed care setting. Journal of Consulting and Clinical Psychology 2004;**72**(4):625–35.

**Al Kubaisy 1992 {published and unpublished data}**

*Al Kubaisy T, Marks I, Logsdail S, Marks MP, Lovell K, Sungur M, et al. Role of exposure homework in phobia reduction: a controlled study. Behavior Therapy 1992;**23**: 599–621.

Park JM, Mataix-Cols D, Marks IM, Ngamthipwatthana T, Marks M, Araya R, et al. Two-year follow-up after a randomised controlled trial of self- and clinician- accompanied exposure for phobia/panic disorders. British Journal of Psychiatry 2001;**178**:543–8.

**Arch 2012 {published data only}**

*Arch JJ, Eifert GH, Davies C, Plumb VJC, Rose RD, Craske MG. Randomized clinical trial of cognitive behavioral therapy (CBT) versus acceptance and commitment therapy (ACT) for mixed anxiety disorders. Journal of Consulting and Clinical Psychology 2012;**80**(5): 750–65.

Arch JJ, Wolitzky-Taylor KB, Eifert GH, Craske MG. Longitudinal treatment mediation of traditional cognitive behavioral therapy and acceptance and commitment therapy for anxiety disorders. Behaviour Research and Therapy 2012; **50**:469–78.

**Arntz 2002 {published data only (unpublished sought but not used)}**

Arntz A. Cognitive therapy versus interoceptive exposure as treatment of panic disorder without agoraphobia. Behaviour Research and Therapy 2002;**40**(3):325–41.

**Barlow 1989 {published data only (unpublished sought but not used)}**

Barlow DH, Brown TA, Craske MG, Rapee RM, Antony MM. Treatment of panic disorder: follow-up and mechanisms of action. 25th Annual Meeting of the Association for the Advancement of Behavior Therapy.

New York, 1991.

*Barlow DH, Craske MG, Cerny JA, Klosko JS. Behavioral treatment of panic disorder. Behavior Therapy 1989;**20**(2): 261–82. Brown TA, Antony MM, Barlow DH. Diagnostic comorbidity in panic disorder: effect on treatment outcome and course of comorbid diagnoses following treatment. Journal of Consulting and Clinical Psychology 1995;**63**(3): 408–18.

Brown TA, Barlow DH. Long-term outcome in cognitive- behavioral treatment of panic disorder: clinical predictors and alternative strategies for assessment. Journal of Consulting and Clinical Psychology 1995;**63**(5):754–65.

Craske MG, Brown TA, Barlow DH. Behavioral treatment of panic disorder: a two-year follow-up. Behavior Therapy 1991;**22**(3):289–304.

Margraf J, Barlow DH, Clark DM, Telch MJ. Psychological treatment of panic: work in progress on outcome, active ingredients, and follow-up. Behavior Research and Therapy 1993;**31**(1):1–8.

**Beck 1987 {published data only (unpublished sought but not used)}**

*Beck AT. Cognitive approaches to panic disorder: theory and therapy. In: Rachman S, Maseck J editor(s). Panic: Psychological Perspectives. Hillsdale, NJ: L. Erlbaum Associates, 1988:91–109.

Sokol-Kessler L, Beck AT. Cognitive treatment of panic disorders. 140th Annual Meeting of the American Psychiatric Association. Chicago, May 1987.

**Beck 1992 {published data only (unpublished sought but not used)}**

Beck AT, Sokol L, Clark DA, Berchick R, Wright F. A crossover study of focused cognitive therapy for panic disorder. American Journal of Psychiatry 1992;**149**(6):778–83.

**Beck 1994 {published data only}**

*Beck JG, Stanley MA, Baldwin LE, Deagle EA, Averill PM. Comparison of cognitive therapy and relaxation training for panic disorder. Journal of Consulting and Clinical Psychology 1994;**62**(4):818–26.

Stanley MA, Beck JG, Averill PM, Baldwin LE, Deagle EA, Stadler JG. Patterns of change during cognitive behavioral treatment for panic disorder. Journal of Nervous and Mental Disease 1996;**184**(9):567–72.

**Bell 2012 {published data only (unpublished sought but not used)}**

Bell C. Computerised cognitive behaviour therapy (CBT) for the treatment of anxiety and effects on neuropsychological functioning and hormone levels. Australian & New Zealand Clinical Trials Registry 2006; ACTRN12606000349549.

*Bell CJ, Colhoun HC, Carter FA, Frampton CM. Effectiveness of computerised cognitive behaviour therapy for anxiety disorders in secondary care. Australian and New Zealand Journal of Psychiatry 2012;**46**(7):630-640.

**Berger 2014 {published data only (unpublished sought but not used)}**

Berger T, Boettcher J, Caspar F. Internet-based guided self-help for several anxiety disorders: A randomized controlled trial comparing a tailored with a standardized disorder-specific approach. Psychotherapy: theory, research and practice 2014;**51**(2):207-219.

**Bergstrom 2010 {published data only}**

Lindefors N. Internet-versus group-administered cognitive behaviour therapy for panic disorder. ClinicalTrials.gov 2009 ; NCT00845260

*Bergstrom J, Andersson G, Ljotsson B, Ruck C, Andreewitch S, Karlsson A, Carlbring P, Andersson E, Lindefors N. Internet-versus group-administered cognitive behaviour therapy for panic disorder in a psychiatric setting: a randomised trial. BMC Psychiatry 2010;**10**:54

**Botella 2004 {published and unpublished data}**

Botella C, Garcia-Palacios A, Villa H, Banos RM, Quero S, Alcaniz M, et al. Virtual reality exposure in the treatment of panic disorder and agoraphobia: a controlled study. Clinical Psychology and Psychotherapy 2007;**14**(3):164–75.

*Botella C, Villa H, Garcia Palacios A, Quero S, Banos RM, Alcaniz M. The use of VR in the treatment of panic disorders and agoraphobia. Studies in Health Technology & Informatics 2004;**99**:73–90.

**Bouchard 1996 {published data only}**

Bouchard S, Gauthier J, Laberge B, French D, Pelletier MH, Godbout C. Exposure versus cognitive restructuring in the treatment of panic disorder with agoraphobia. Behaviour Research and Therapy 1996;**34**(3):213-24

**Brown 1997 {published data only (unpublished sought but not used)}**

Brown GK, Beck AT, Newman CF, Beck JS, Tran GQ. A comparison of focused and standard cognitive therapy for panic disorder. Journal of Anxiety Disorders 1997;**11**(3):329–45.

**Burke 1997 {published data only}**

Burke M, Drummond LM, Johnston DW. Treatment choice for agoraphobic women: exposure or cognitive- behaviour therapy? British Journal of Clinical Psychology 1997;**36**(Pt 3):409–20.

**Carlbring 2001 {published data only (unpublished sought but not used)}**

Carlbring P, Westling BE, Ljungstrand P, Ekselius L, Andersson G. Treatment of panic disorder via the Internet: a randomized trial of a self-help program. Behavior Therapy 2001;**32**(4):751-64

**Carlbring 2003 {published data only}**

Carlbring P, Ekselius L, Andersson G. Treatment of panic disorder via the Internet: a randomized trial of CBT vs. applied relaxation. Journal of Behavior Therapy and Experimental Psychiatry 2003;**34**(2):129-40

**Carlbring 2004 {published data only}**

Carlbring P, Nilsson-Ihrfelt E, Waara J, Kollenstam C, Burman M, Kaldo V, et al. Treatment of panic disorder: Live therapy vs. self-help via the internet. 32nd Congress of the British Association for Behavioural and Cognitive Psychotherapies (jointly with the European Association of Behavioural and Cognitive Therapies); 2004 September 7-11; Manchester 2004:166

*Carlbring P, Nilsson-Ihrfelt E, Waara J, Kollenstam C, Buhrman M, Kaldo V, et al. Treatment of panic disorder: Live therapy vs. self-help via the Internet. Behaviour Research and Therapy 2005;**43**(10):1321-33

Andersson G, Carlbring P, Grimlund A. Predicting treatment outcome in internet versus face to face treatment of panic disorder. Computers in Human Behavior 2008;**24**(5):1790-1801

**Carlbring 2006 {published data only}**

*Carlbring P, Bohman S, Brunt S, Buhrman M, Westling BE, Ekselius L, Andersson G. Remote treatment of panic disorder: a randomized trial of internet-based cognitive behavior therapy supplemented with telephone calls. American Journal of Psychiatry 2006;**163**(12):2119-2125

Carlbring P, Maurin T, Sjomark J, Maurin L, Westling BE, Ekselius L, et al. All at once or one at a time? A randomized controlled trial comparing two ways to deliver bibliotherapy for panic disorder. Cognitive Behaviour Therapy 2011;**40**(3):228-235

**Carter 2003 {published data only}**

Carter MM, Sbrocco T, Gore KL, Marin NW, Lewis EL. Cognitive-behavioral group therapy versus a wait-list control in the treatment of African American women with panic disorder. Cognitive Therapy and Research 2003;**27**(5): 505–18.

**Choi 2003 {published data only}**

Choi YH. The development and the effects of experiential cognitive therapy for the treatment of panic disorder with agoraphobia. 156th Annual Meeting of the American Psychiatric Association, May 17-22, San Francisco (CA) 2003; NR813

*Choi YH, Vincelli F, Riva G, Wiederhold BK, Lee JH, Park KH. Effects of group experiential cognitive therapy for the treatment of panic disorder with agoraphobia. CyberPsychology and Behavior 2005;**8**(4):387-93

**Clark 1994 {published data only}**

Clark DM, Salkovskis PM, Hackmann A, Middleton H, Anastasiades P, Gelder M. A comparison of cognitive therapy, applied relaxation and imipramine in the treatment of panic disorder. British Journal of Psychiatry 1994;**164**(6): 759–69.

**Clark 1999 {published data only}**

Clark DM, Salkovskis PM, Hackmann A, Wells A, Ludgate J, Gelder M. Brief cognitive therapy for panic disorder: a randomized controlled trial. Journal of Consulting and Clinical Psychology 1999;**67**(4):583–9.

**Cottraux 2009 {published data only (unpublished sought but not used)}**

Cottraux J. A comparative controlled study of virtual reality therapy and cognitive behavior therapy in panic disorder with agoraphobia. https://clinicaltrials.gov/ct2/show/ NCT00129610 2005 (accessed 16 March 2015).

Lambrey S, Jouvent R, Allilaire JF, Pelissolo A. Virtual reality therapies in the treatment of phobic disorders [French]. Annales Medico-Psychologiques 2010;**168**(1):44–6.

*Pelissolo A, Zaoui M, Aguayo G, Yao SN, Roche S, Ecochard R, et al. Virtual reality exposure therapy versus cognitive behavior therapy for panic disorder with agoraphobia: a randomized comparison study. Journal of CyberTherapy and Rehabilitation 2012;**5**(1):35–43.

Pull C, Pelissolo A, Zaoui M, et al. A randomized controlled study of virtual reality exposure therapy and cognitive- behavior therapy in panic disorder with agoraphobia. Cyberpsychology & Behavior (Abstracts from CyberTherapy 14 Designing the Future of Healthcare, June 21-23, 2009, Lago Maggiore, Verbania, Italy) 2009;**12**(5):647.

**Craske 1995 {published data only}**

Craske MG, Maidenberg E, Bystritsky A. Brief cognitive- behavioral versus nondirective therapy for panic disorder. Journal of Behavior Therapy and Experimental Psychiatry 1995;**26**(2):113–20.

**Craske 1997 {published data only}**

Craske MG, Rowe M, Lewin M, Noriega Dimitri R. Interoceptive exposure versus breathing retraining within cognitive-behavioural therapy for panic disorder with agoraphobia. British Journal of Clinical Psychology 1997;**36**:85-99

**Craske 2003 {published data only}**

Craske MG, DeCola JP, Sachs AD, Pontillo DC. Panic control treatment for agoraphobia. Journal of Anxiety Disorders 2003;**17**(3):321-33

**Craske 2005a {published data only}**

*Craske MG, Lang AJ, Aikins D, Mystkowski JL. Cognitive behavioral therapy for nocturnal panic. Behavior Therapy 2005;**36**(1):43–54.

Tsao JC, Mystkowski JL, Zucker BG, Craske MG. Impact of cognitive-behavioral therapy for panic disorder on comorbidity: a controlled investigation. Behaviour Research and Therapy 2005;**43**(7):959–70.

**Creager Berger 2001 {published data only (unpublished sought but not used)}**

*Creager Berger B. The treatment of panic disorder: a comparative study between breathing retraining and cognitive behavioral therapy. Dissertation Abstracts International 2001;**61**(8b)

Creager Berger B, Gevirtz R. The treatment of panic disorder: a comparison between breathing retraining and cognitive behavioral therapy. Biological Psychology 2001;**56** (1):78–9

**De Beurs 1995 {published data only}**

van Balkom A, de Beurs E, van Dyck R, Lange A. Two-year follow-up after treatment of panic disorder with agoraphobia with fluvoxamine, placebo, psychological panic management combined with exposure and of exposure in vivo alone. 8th ECNP (European College of Neuropsychopharmacology) Congress, Venice, Italy 1995

*de Beurs E, van Balkom AJ, Lange A, Koele P, van Dyck R. Treatment of panic disorder with agoraphobia: comparison of fluvoxamine, placebo, and psychological panic management combined with exposure and of exposure in vivo alone . American Journal of Psychiatry 1995;**152**(5):683-91

de Beurs E, van Dyck R, Lange A, van Balkom AJ. Perceived upbringing and its relation to treatment outcome in agoraphobia. Clinical Psychology and Psychotherapy 1995;**2**(2):78-85

van Balkom AJ, de Beurs E, Koele P, Lange A, van Dyck R. Long-term benzodiazepine use is associated with smaller treatment gain in panic disorder with agoraphobia. Journal of Nervous and Mental Disease 1996;**184**(2):133-5

van Balkom AJ, de Beurs E, van Dyck R, Lange A. Two-year follow-up after treatment of panic disorder with agoraphobia. XXth Collegium Internationale Neuropsychopharmacologicum, Melbourne, Australia 1996

de Beurs E, van Balkom AJ, Van Dyck R, Lange A. Long-term outcome of pharmacological and psychological treatment for panic disorder with agoraphobia: a 2-year naturalistic follow-up. Acta Psychiatrica Scandinavica 1999;**99**(1):59-67

**De Ruiter 1989 {published data only}**

Rijken H, Kraaimaat F, de Ruiter C, Garssen B. A follow-up study on short-term treatment of agoraphobia. Behaviour Research and Therapy 1992;**30**(1):63–6.

*de Ruiter C, Ryken H, Garssen B, Kraaimaat F. Breathing retraining, exposure and a combination of both, in the treatment of panic disorder with agoraphobia. Behaviour Research and Therapy 1989;**27**(6):647–55.

**Dow 2000 {published data only (unpublished sought but not used)}**

Casey LM, Newcombe PA, Oei TP. Cognitive mediation of panic severity: the role of catastrophic misinterpretation of bodily sensations and panic self-efficacy. Cognitive Therapy and Research 2005;**29**(2):187–200.

Dow MG, Kenardy JA, Johnston DW, Newman MG, Taylor CB, Thomson A. Prognostic indices with brief and standard CBT for panic disorder: I. Predictors of outcome. Psychological Medicine 2007;**37**(10):1493–502.

Kenardy J, Robinson S, Dob R. Cognitive behaviour therapy for panic disorder: long-term follow up. Cognitive Behaviour Therapy 2005;**34**(2):75–8.

*Kenardy JA, Dow MG, Johnston DW, Newman MG, Thomson A, Taylor CB. A comparison of delivery methods of cognitive-behavioral therapy for panic disorder: an international multicenter trial. Journal of Consulting and Clinical Psychology 2003;**71**(6):1068–75.

**Dreessen 1994 {published data only (unpublished sought but not used)}**

Arntz A, van den Hout M. Psychological treatments of panic disorder without agoraphobia: cognitive therapy versus applied relaxation. Behaviour Research and Therapy 1996;**34**(2):113–21.

**Emmelkamp 1986 {published data only (unpublished sought but not used)}**

Emmelkamp PM, Brilman E, Kuiper H, Mersch PP. The treatment of agoraphobia. A comparison of self- instructional training, rational emotive therapy, and exposure in vivo. Behavior Modification 1986;**10**:37–53.

**Erickson 2003 {published data only (unpublished sought but not used)}** Erickson DH, Janeck AS, Tallman K. A cognitive-behavioral group for patients with various anxiety disorders. Psychiatric Services 2007;**58**(9):1205–11.

Erickson DH, Janeck AS, Tallman K. Group CBT for heterogeneous anxiety disorders. 34th Annual Conference of the British Association for Behavioural and Cognitive Psychotherapies. Warwick, 19–21 July 2006. Erickson DH, Janeck AS, Tallman K. Group CBT for heterogeneous anxiety disorders: preliminary evidence for effectiveness. 31st Annual Conference of the British Association for Behavioural and Cognitive Psychotherapies. York, 16–19 July 2003.

**Gloster 2010 {published data only}**

Arolt V, Zwanziger P, Strohle A, Hamm A, Gerlach A, Kircher T, et al. The research network PANIC-NET: improving the treatment of panic disorder - From a better understanding of fear circuit mechanisms to more effective psychological treatment and routine care. Psychotherapie Psychosomatik Medizinische Psychologie 2009;**59**:124–31.

Emmrich A, Beesdo-Baum K, Gloster AT, Knappe S, Hfler M, Arolt V, et al. Depression does not affect the treatment outcome of CBT for panic and agoraphobia: results from a multicenter randomized trial. Psychotherapy and Psychosomatics 2012;**81**(3):161–72.

Gloster AT, Hauke C, Hofler M, Einsle F, Fydrich T, Hamm A, et al. Long-term stability of cognitive behavioral therapy effects for panic disorder with agoraphobia: a two-year follow-up study. Behaviour Research and Therapy 2013;**51** (12):830–9.

Gloster AT, Klotsche J, Gerlach AL, Hamm A, Strohle A, Gauggel S, et al. Timing matters: change depends on the stage of treatment in cognitive behavioral therapy for panic disorder with agoraphobia. Journal of Consulting and Clinical Psychology 2014;**82**(1):141–53.

Gloster AT, Wittchen HU, Einsle F, Hofler M, Lang T, Helbig-Lang S, et al. Mechanism of action in CBT (MAC): methods of a multi-center randomized controlled trial in 369 patients with panic disorder and agoraphobia. European Archives of Psychiatry & Clinical Neuroscience 2009;**259** (Suppl 2):S155–66.

Gloster AT, Wittchen HU, Einsle F, Lang T, Helbig-Lang S, Fydrich T, et al. Correction to Gloster et al. (2011). Journal of Consulting and Clinical Psychology 2011;**79**(5):652.

*Gloster AT, Wittchen HU, Einsle F, Lang T, Helbig- Lang S, Fydrich T, et al. Psychological treatment for panic disorder with agoraphobia: a randomized controlled trial to examine the role of therapist-guided exposure in situ in CBT. Journal of Consulting and Clinical Psychology 2011;**79** (3):406–20.

Kircher T, Arolt V, Jansen A, Pyka M, Reinhardt I, Kellermann T, et al. Effect of cognitive-behavioral therapy on neural correlates of fear conditioning in panic disorder. Biological Psychiatry 2013;**73**(1):93–101.

Lang T, Helbig-Lang S, Gloster AT, et al. Effects of therapist-guided exposure in CBT for panic disorder and agoraphobia [Effekte therapeutenbegleiteter versus patientengeleiteter Exposition bei Panikstörung mit Agoraphobie]. Zeitschrift für klinische Psychologie und Psychotherapie 2012;**41**(2):114–24.

Wittchen HU, Lang T. Improving cognitive behavioural therapy for panic by identifying the active ingredients and understanding the mechanisms of action: a multicentre study. http://www.isrctn.com/ISRCTN80046034 2007 (accessed 16 March 2015).

**Goldstein 2000 {published data only (unpublished sought but not used)}**

Goldstein AJ, De Beurs E, Chambless DL, Wilson KA. EMDR for panic disorder with agoraphobia: comparison with waiting list and credible attention-placebo control conditions. Journal of Consulting and Clinical Psychology 2000;**68**(6):947–56.

**Gould 1993 {published data only}**

Gould R, Clum GA, Shapiro D, Weaver T, Blalock J. Evidence for a self-help coping approach for treating panic disorder. Annual Meeting of the Southeastern Psychological Association. New Orleans, LA, 1991.

*Gould RA, Clum GA, Shapiro D. The use of bibliotherapy in the treatment of panic: a preliminary investigation. Behavior Therapy 1993;**24**:241–52.

**Gould 1995 {published data only}**

Gould RA, Clum GA. Self-help plus minimal therapist contact in the treatment of panic disorder: a replication and extension. Behavior Therapy 1995;**26**:533-46

**Griegel 1995 {published data only}**

Griegel LE. Breathing retraining in panic disorder: physiological mechanisms or perceived controllability. Dissertation Abstracts International: Section B: The Sciences and Engineering 1995;**55**(9):4120.

**Hazen 1996 {published data only (unpublished sought but not used)}**

Hazen AL, Walker JR, Eldridge GD. Anxiety sensitivity and treatment outcome in panic disorder. Anxiety 1996;**2**(1): 34–9.

Walker JG, Eldridge A, Hazen, Rallo J, O’Riordan J, Frankel S. Evaluation of a self-help book and workbook for treatment of panic disorder used independently, in a self- help group, or a professional group [Thesis]. University of Manitoba 1996.

**Hecker 2004 {published data only (unpublished sought but not used)}**

Hecker JE, Losee MC, Roberson-Nay R, Maki K. Mastery of Your Anxiety and Panic and brief therapist contact in the treatment of panic disorder. Journal of Anxiety Disorders 2004;**18**(2):111-26

**Helbig 2009 {published and unpublished data}**

Helbig S, Lang T, Swendsen J, Hoyer J, Wittchen HU. Feasibility, compliance and information content of an ecological momentary assessment approach in patients with panic disorder and agoraphobia. Zeitschrift fur Klinische Psychologie und Psychotherapie: Forschung und Praxis 2009;**38**(2):108-17

**Hendriks 2010 {published data only}**

Hendriks GJ. The efficacy of paroxetine and cognitive-behavioural therapy in the treatment of late-life panic disorder: a randomized controlled trial. http:// www.trialregister.nl/ 2007 [NTR1144]

Hendriks GJ, Keijsers GPJ, Kampman M, Hoogduin CAL, Oude Voshaar RC. Predictors of outcome of pharmacological and psychological treatment of late-life panic disorder with agoraphobia. International Journal of Geriatric Psychiatry 2012;**27**(2):146–50.

*Hendriks GJ, Keijsers GPJ, Kampman M, Oude Voshaar RC, Verbraak M, Broekman TG, et al. A randomized controlled study of paroxetine and cognitive-behavioural therapy for late-life panic disorder. Acta Psychiatrica Scandinavica 2010;**122**(1):11–9.

**Hoffart 1995 {published data only}**

*Hoffart A. A comparison of cognitive and guided mastery therapy of agoraphobia. Behaviour Research and Therapy 1995;**33**(4):423–34.

Hoffart A. Cognitive and guided mastery therapy of agoraphobia: long-term outcome and mechanisms of change. Cognitive Therapy and Research 1998;**22**(3): 195–207.

Hoffart A. Cognitive mediators of situational fear in agoraphobia. Journal of Behavior Therapy and Experimental Psychiatry 1995;**26**(4):313–20.

Hoffart A. Interpersonal problems among patients suffering from panic disorder with agoraphobia before and after treatment. British Journal of Medical Psychology 1997;**70**(2): 149–57.

Hoffart A, Hedley LM. Personality traits among panic disorder with agoraphobia patients before and after symptom-focused treatment. Journal of Anxiety Disorders 1997;**11**(1):77–87.

Hoffart A, Hedley LM, Thornes K, Larsen SM, Friis S. Therapists’ emotional reactions to patients as a mediator in cognitive behavioural treatment of panic disorder with agoraphobia. Cognitive Behaviour Therapy 2006;**35**(3): 174–82.

**Ito 1996 {published data only}**

Ito LM, Noshirvani H, Başoğlu M, Marks IM. Does exposure to internal cues enhance exposure to external cues in agoraphobia with panic? A pilot controlled study of self-exposure. Psychotherapy & Psychosomatics 1996;**65**(1):24-28

**Karekla 2004 {published data only (unpublished sought but not used)}**

*Karekla M. A Comparison Between Acceptance Enhanced Cognitive Behavioral and Panic Control Treatment for Panic Disorder [Doctoral dissertation]. University at Albany, State University of New York, 2004.

Karekla M. A comparison between acceptance-enhanced panic control treatment and panic control treatment for panic disorder. Dissertation Abstracts International 2005;**65** (9b):4835.

Levitt Jill T, Karekla M. Integrating acceptance and mindfulness with cognitive behavioral treatment for panic disorder. Acceptance and mindfulness-based approaches to anxiety: conceptualization and treatment. Springer US, 2005:165–88.

**Kiropoulos 2006 {published data only}**

Kiropoulos LA, Klein B, Austin DW, Pier C, Mitchell J. Is internet-based CBT for panic disorder and agoraphobia as effective as face-to-face CBT? 29th Australian Association for Cognitive and Behaviour Therapy Annual Conference, 2006 October 18-23

*Kiropoulos LA, Klein B, Austin DW, Gilson K, Pier C, Mitchell J, Ciechomski L. Is internet-based CBT for panic disorder and agoraphobia as effective as face-to-face CBT? Journal of Anxiety Disorders 2008;**22**(8):1273-84

**Klein 2001 {published data only (unpublished sought but not used)}**

Klein B, Richards JC. A brief internet-based treatment for panic disorder. Behavioural & Cognitive Psychotherapy 2001;**29**(1):113-7

**Klosko 1988 {published data only}**

Klosko JS. Comparison of alprazolam and cognitive- behavior therapy in treatment of panic disorder. Dissertation Abstracts International 1988;**49**(5-b):1945.

*Klosko JS, Barlow DH, Tassinari R, Cerny JA. A comparison of alprazolam and behavior therapy in treatment of panic disorder. Journal of Consulting and Clinical Psychology 1990; **58**(1):77–84.

Klosko JS, Barlow DH, Tassinari RB, Cerny JA. Comparison of alprazolam and cognitive-behavior therapy in the treatment of panic disorder. A preliminary report. Panic and Phobias: Treatment and Variables Affecting Course and Outcome. Springer-Verlag, 1988.

**Korrelboom 2013 {published data only}**

Korrelboom K, Peeters S, Blom S, Huijbrechts I. Competitive memory training (COMET) for panic and applied relaxation (AR) are equally effective in the treatment of panic in panic-disordered patients. Journal of Contemporary Psychotherapy 2014;**44**(3):183–90. [DOI: 10.1007/s10879-013-9259-3]

**Lidren 1994 {published data only}**

Lidren DM. The differential efficacy of self-help and group therapy in the treatment of panic disorder and agoraphobia: a treatment comparison study. Dissertation Abstracts International 1994;**54**(12-b):6465.

*Lidren DM, Watkins PL, Gould RA, Clum GA, Asterino M, Tulloch HL. A comparison of bibliotherapy and group therapy in the treatment of panic disorder. Journal of Consulting and Clinical Psychology 1994;**62**(4):865–9.

**Malbos 2011 {published and unpublished data}**

*Malbos E, Rapee RM, Kavakli M. A controlled study of agoraphobia and the independent effect of virtual reality exposure therapy. Australian and New Zealand Journal of Psychiatry 2013;**47**(2):160–8.

Malbos E, Rapee RM, Kavakli M. Isolating the effect of virtual reality based exposure therapy for agoraphobia: a comparative trial. Studies in Health Technology and Informatics 2011;**167**:45–50.

**Marchione 1987 {published data only}**

Marchione KE, Michelson L, Greenwald M, Dancu C. Cognitive behavioral treatment of agoraphobia. Behaviour Research and Therapy 1987;**25**(5):319–28.

**Meulenbeek 2008 {published and unpublished data}**

Meulenbeek P, Spinhoven P, Smit F, van Balkom A, Cuijpers P. Cognitive mediation of panic reduction during an early intervention for panic. Acta Psychiatrica Scandinavica 2010; **122**(1):20–9.

*Meulenbeek P, Willemse G, Smit F, van Balkom A, Spinhoven P, Cuijpers P. Early intervention in panic: pragmatic randomised controlled trial. British Journal of Psychiatry 2010;**196**:326–31.

Meulenbeek P, Willemse G, Smit F, van Balkom A, Spinhoven P, Cuijpers P. Early intervention in panic: pragmatic randomised controlled trial: Erratum. British Journal of Psychiatry 2010;**196**(6):497.

Meulenbeek P, Willemse G, Smit F, van Balkom A, Spinhoven P, Cuijpers P. Early intervention in panic: randomized controlled trial and cost-effectiveness analysis. Trials 2008;**9**:67.

Willemse G. Prevention of panic disorder: a randomised clinical trial adjoining cost-effectiveness study. http:// www.isrctn.com/ISRCTN33407455 2005 (accessed 16 March 2015).

**Meuret 2008 {published data only (unpublished sought but not used)}**

Meuret AE, Wilhelm FH, Ritz T, Roth WT. Feedback of end-tidal pCO2 as a therapeutic approach for panic disorder. Journal of Psychiatric Research 2008;**42**(7):560–8.

**Meuret 2010 {published data only (unpublished sought but not used)}**

Meuret AE, Hofmann SG, Rosenfield D. Catastrophic appraisal and perceived control as moderators of treatment response in panic disorder. International Journal of Cognitive Therapy 2010;**3**(3):262–77.

*Meuret AE, Rosenfield D, Seidel A, Bhaskara L, Hofmann SG. Respiratory and cognitive mediators of treatment change in panic disorder: evidence for intervention specificity. Journal of Consulting and Clinical Psychology 2010;**78**(5):691–704. Meuret AE, Seidel A, Rosenfield B, Hofmann SG, Rosenfield D. Does fear reactivity during exposure predict panic symptom reduction? Journal of Consulting and Clinical Psychology 2012;**80**(5):777–85.

**Meyerbroker 2011 {published and unpublished data}**

Emmelkamp PMG. Virtual reality exposure therapy in agoraphobic participants. https://clinicaltrials.gov/ct2/ show/NCT00734370 2008 (accessed 16 March 2015).

Meyerbroker K, Morina N, Kerkhof G, Emmelkamp PM. Virtual reality exposure treatment of agoraphobia: a comparison of computer automatic virtual environment and head-mounted display. Studies in Health Technology & Informatics 2011;**167**:51–6.

*Meyerbroker K, Morina N, Kerkhof G, Emmelkamp PMG. Virtual reality exposure therapy does not provide any additional value in agoraphobic patients: a randomized controlled trial. Psychotherapy and Psychosomatics 2013;**82** (3):170–6.

Meyerbroker K, Morina N, Kerkhof G, Emmelkamp PMG. Virtual reality exposure treatment of agoraphobia: a comparison of computer automatic virtual environment and head-mounted display. Annual Review of CyberTherapy and Telemedicine 2011;**9**(1):41–5.

**Michelson 1985 {published data only}**

Michelson L, Mavissakalian M, Marchione K. Cognitive and behavioral treatments of agoraphobia: Clinical, behavioral, and psychophysiological outcomes. Journal of Consulting and Clinical Psychology 1985;**53**(6):913-25

Michelson L. Treatment consonance and response profiles in agoraphobia: the role of individual differences in cognitive, behavioral and physiological treatments. Behaviour Research and Therapy 1986;**24**(3):263-75

Schwartz RM, Michelson L. States-of-mind model: Cognitive balance in the treatment of agoraphobia. Journal of Consulting and Clinical Psychology 1987;**55**(4):557-65

Michelson L, Mavissakalian M, Marchione K. Cognitive, behavioral, and psychophysiological treatments of agoraphobia: a comparative outcome investigation. Behavior Therapy 1988;**19**:97-120

Michelson L, Mavissakalian M, Marchione K, Ulrich RF, Marchione N, Testa S. Psychophysiological outcome of cognitive behavioral and psychophysiologically-based treatments of agoraphobia. Behaviour Research and Therapy 1990;**28**(2):127-39

Michelson LK, Bellanti CJ, Testa SM, Marchione N. The relationship of attributional style to agoraphobia severity, depression, and treatment outcome. Behaviour Research and Therapy 1997;**35**(12):1061-73

**Milrod 2015 {published and unpublished data}**

Milrod B, Leon AC, Fishman B, Barber JP, Chambless D. Dynamic treatment vs. CBT for panic disorder. ClinicalTrials.gov 2006 ; [NCT00353470]

Zilcha-Mano S, McCarthy KS, Dinger U, Chambless DL, Milrod BL, Kunik L and Barber JP. Are there subtypes of panic disorder? An interpersonal perspective. Journal of Consulting and Clinical Psychology 2015;**83**(5):938-50

*Milrod B, Chambless DL, Gallop R, Busch FN, Schwalberg M, McCarthy KS, Gross C, Sharpless BA, Leon AC and Barber JP. Psychotherapies for panic disorder: a tale of two sites. The Journal of clinical psychiatry 2015; Epub 2015 Jun 9

**Muncy 1991 {published data only}**

Muncy SM. Panic: a comparison of four treatment methods. Dissertation Abstracts International 1991;**51**(12b, pt 1):6115.

**Nordin 2010 {published data only}**

Nordin S, Carlbring P, Cuijpers P, Andersson G. Expanding the limits of bibliotherapy for panic disorder: randomized trial of self-help without support but with a clear deadline. Behavior Therapy 2010;**41**(3):267-76

**Ost 1993 {published data only (unpublished sought but not used)}**

Ost LG, Westling BE, Hellstrom K. Applied relaxation, exposure in vivo and cognitive methods in the treatment of

panic disorder with agoraphobia. Behaviour Research and Therapy 1993;**31**(4):383–94.

**Ost 1995 {published data only}**

Ost LG, Westling BE. Applied relaxation vs cognitive behavior therapy in the treatment of panic disorder. Behaviour Research and Therapy 1995;**33**(2):145–58.

**Ost 2004 {published data only (unpublished sought but not used)}**

Ost LG, Thulin U, Ramnero J. Cognitive behavior therapy vs exposure in vivo in the treatment of panic disorder with agoraphobia. Behaviour Research and Therapy 2004;**42**(10): 1105–27.

Ramnero J, Ost LG. Panic and avoidance in panic disorder with agoraphobia: clinical relevance of change in different aspects of the disorder. Journal of Behavior Therapy and Experimental Psychiatry 2007;**38**(1):29–39.

**Perez-Ara 2010 {published data only}**

Pérez-Ara MA, Quero S, Botella C, Baños R, Andreu-Mateu S, García-Palacios A, Bretón-López J. Virtual reality interoceptive exposure for the treatment of panic disorder and agoraphobia. Studies in Health Technology & Informatics 2010;**154**:77-81

**Petterson 1996 {published data only}**

Petterson K, Cesare S. Panic disorder: a cognitive- behavioural approach to treatment. Counselling Psychology Quarterly 1996;**9**(2):191–201.

**Power 2000 {published data only}**

Sharp DM, Power KG, Swanson V. Therapist contact in cognitive behavioural therapy for panic disorder and agoraphobia in primary care: A randomised controlled trial. 28th Annual Conference of the British Association for Behavioural and Cognitive Psychotherapies; 2000 July 19-22, London

Sharp DM, Power KG, Swanson V. Reducing therapist contact in cognitive behaviour therapy for panic disorder and agoraphobia in primary care: global measures of outcome in a randomised controlled trial. British Journal of General Practice 2000;**50**(461):963-8

Power KG, Sharp DM, Swanson V, Simpson RJ. Therapist contact in cognitive behaviour therapy for panic disorder and agoraphobia in primary care. Clinical Psychology and Psychotherapy 2000;**7**(1)37-46

**Rees 1999 {published data only}**

Rees CS, Richards JC, Smith LM. The efficacy of information-giving in cognitive-behavioural treatment for panic disorder. Behaviour Change 1999;**16**(3):175–81.

**Reinecke 2013 {published data only}**

Reinecke A, Waldenmaier L, Cooper MJ, Harmer CJ. Changes in automatic threat processing precede and predict clinical changes with exposure-based cognitive-behavior therapy for panic disorder. Biological Psychiatry 2013;**73** (11):1064–70.

**Richards 2006 {published data only}**

Richards JC, Klein B, Austin DW. Internet cognitive behavioural therapy for panic disorder: does the inclusion of stress management information improve end-state functioning? Clinical Psychologist 2006;**10**(1):2-15

**Salkovskis 1999 {published data only}**

Salkovskis PM, Clark DM, Hackmann A, Wells A, Gelder MG. An experimental investigation of the role of safety- seeking behaviours in the maintenance of panic disorder with agoraphobia. Behaviour Research and Therapy 1999;**37** (6):559–74.

Salkovskis PM, Hackmann A, Wells A, Gelder MG, Clark DM. Belief disconfirmation versus habituation approaches to situational exposure in panic disorder with agoraphobia: a pilot study. Behaviour Research and Therapy 2007;**45**(5): 877–85.

**Schmidt 1997a {published data only}**

Schmidt NB, Trakowski JH, Staab JP. Extinction of panicogenic effects of a 35% CO2 challenge in patients with panic disorder. Journal of Abnormal Psychology 1997; **106**(4):630–8.

**Schmidt 1997b {published data only (unpublished sought but not used)}**

*Schmidt NB, Staab JP, Trakowski JH Jr, Sammons M. Efficacy of a brief psychosocial treatment for panic disorder in an active duty sample: implications for military readiness. Military Medicine 1997;**162**(2):123–9.

Schmidt NB, Woolaway-Bickel K, Trakowski J, Santiago H, Storey J, Koselka M, et al. Dismantling cognitive- behavioral treatment for panic disorder: questioning the utility of breathing retraining. Journal of Consulting and Clinical Psychology 2000;**68**(3):417–24.

**Schmidt 2000 {published data only}**

Schmidt NB, Woolaway-Bickel K, Trakowski J, Santiago H, Storey J, Koselka M, Cook J. Dismantling cognitive-behavioral treatment for panic disorder: questioning the utility of breathing retraining. Journal of Consulting and Clinical Psychology 2000;**68**(3)417-24

**Scott 1995 {published data only (unpublished sought but not used)}**

Scott MJ, Stradling SG, Greenfield TA. The efficacy of brief group cognitive therapy programmes for anxiety and depression, and the relevance of a personality disorder diagnosis. World Congress of Behavioural and Cognitive Therapies; 1995 July 11-15. Copenhagen, 1995.

**Sharp 2004 {published data only (unpublished sought but not used)}**

Sharp DM, Power KG, Swanson V. A comparison of the efficacy and acceptability of group versus individual cognitive behaviour therapy in the treatment of panic disorder and agoraphobia in primary care. Clinical Psychology and Psychotherapy 2004;**11**(2):73–82.

**Shear 1994 {published data only}**

Shear MK, Leon AC, Portera L, Klosko J, Cloitre M. Psychoeducation /reflective listening compared to cognitive- behavioural treatment in panic disorder. New Research Program and Abstracts - 144th Annual Meeting of the American Psychiatric Association; 1991 May 11-16. New Orleans, Louisiana, US, 1991.

*Shear MK, Pilkonis PA, Cloitre M, Leon AC. Cognitive behavioral treatment compared with nonprescriptive treatment of panic disorder. Archives of General Psychiatry 1994;**51**(5):395–401.

**Taylor 1982 {published data only (unpublished sought but not used)}**

Taylor CB, Kenigsberg ML, Robinson JM. A controlled comparison of relaxation and diazepam in panic disorder. Journal of Clinical Psychiatry 1982;**43**(10):423–5.

**Telch 1993 {published data only}**

Telch MJ, Lucas JA, Schmidt NB, Hanna HH, LaNae Jaimez T, Lucas RA. Group cognitive-behavioral treatment of panic disorder. Behaviour Research and Therapy 1993;**31** (3):279–87.

**Tyrer 1988 {published data only (unpublished sought but not used)}**

Kingdon D, Tyrer P, Seivewright N, Ferguson B, Murphy S. The Nottingham Study of Neurotic Disorder: influence of cognitive therapists on outcome. British Journal of Psychiatry 1996;**169**:93–7.

Klein DF. Nottingham study of neurotic disorder. Lancet 1988;**2**:1015. Knerer G, Byford S, Johnson T, Seivewright H, Tyrer P. The Nottingham study of neurotic disorder: predictors of 12 year costs. Acta Psychiatrica Scandinavica 2005;**112**(3): 224–32.

Seivewright H, Tyrer P, Johnson T. Change in personality status in neurotic disorders. Lancet 2002;**359**:2253–4. Seivewright H, Tyrer P, Johnson T. Prediction of outcome in neurotic disorder: a 5-year prospective study. Psychological Medicine 1998;**28**(5):1149–57.

Seivewright N, Tyrer P, Ferguson B, Murphy S, Johnson T. Longitudinal study of the influence of life events and personality status on diagnostic change in three neurotic disorders. Depression & Anxiety 2000;**11**(3):105–13.

Tyrer P, Seivewright H, Ferguson B, Johnson T. Cold calling in psychiatric follow up studies: is it justified?. Journal of Medical Ethics 2003;**29**(4):238–42.

Tyrer P, Seivewright H, Johnson T. The Nottingham Study of Neurotic Disorder: Predictors of 12-year outcome of dysthymic, panic and generalized anxiety disorder. Psychological Medicine 2004;**34**(8):1385–94.

Tyrer P, Seivewright H, Simmonds S, Johnson T. Prospective studies of cothymia (mixed anxiety-depression): How do they inform clinical practice? European Archives of Psychiatry & Clinical Neuroscience 2001;**251**(Suppl 2): 1153–6.

Tyrer P, Seivewright N, Ferguson B, Murphy S, Darling C, Brothwell J, et al. The Nottingham Study of Neurotic Disorder: relationship between personality status and symptoms. Psychological Medicine 1990;**20**(2):423–31.

Tyrer P, Seivewright N, Ferguson B, Murphy S, Johnson AL. The Nottingham Study of Neurotic Disorder: effect of personality status on response to drug treatment, cognitive therapy and self-help over two years. British Journal of Psychiatry 1993;**162**:219–26.

Tyrer P, Seivewright N, Ferguson B, Tyrer J. The general neurotic syndrome: a coaxial diagnosis of anxiety, depression and personality disorder. Acta Psychiatrica Scandinavica 1992;**85**(3):201–6.

*Tyrer P, Seivewright N, Murphy S, Ferguson B, Kingdon D, Barczak P, et al. The Nottingham Study of Neurotic Disorder: comparison of drug and psychological treatments. Lancet 1988;**2**:235–40.

Tyrer P, Seivewright N, Seivewright H. Long-term outcome of hypochondriacal personality disorder. Journal of Psychosomatic Research 1999;**46**(2):177–85.

**Williams 1996 {published data only (unpublished sought but not used)}**

Williams SL, Falbo J. Cognitive and performance-based treatments for panic attacks in people with varying degrees of agoraphobic disability. Behaviour Research and Therapy 1996;**34**(3):253–64.

**Wims 2010 {published and unpublished data}**

Wims E. The effectiveness of clinician assisted internet based Cognitive Behaviour Therapy for Panic Disorder. ANZCTR 2008; ACTRN12608000548336

*Wims E, Titov N, Andrews G, Choi I. Clinician-assisted Internet-based treatment is effective for panic: A randomized controlled trial. Australian and New Zealand Journal of Psychiatry 2010;**44**(7):599-607

**Wollburg 2011 {published data only}**

*Kim S, Wollburg E, Roth WT. Opposing breathing therapies for panic disorder: a randomized controlled trial of lowering vs raising end-tidal PCO2. Journal of Clinical Psychiatry 2012;**73**(7):931–9.

Roth WT. Breathing regulation training for individuals with panic disorder. Clinicaltrials.gov; NCT00183521 2005.

Wollburg E, Roth WT, Kim S. Effects of breathing training on voluntary hypo- and hyperventilation in patients with panic disorder and episodic anxiety. Applied Psychophysiology & Biofeedback 2011;**36**:81–91.

**Woolaway-Bickel 2008 {published data only}**

Bickel KW. An empirical test of CALM for pd: A computer-administered learning module for panic disorder. Dissertation Abstracts International: The Sciences and Engineering 2008;68(8-B):5558

**References of ongoing studies (assessed as ongoing in november 2015)**

**Andrews 2011a**

Andrews G. Transdiagnostic Program 1 - The Anxiety Program: Internet based education for social phobia, panic disorder (with or without agoraphobia) and generalised anxiety disorder with telephone reminders and online forum support. A randomized controlled study. <https://www.anzctr.org.au/Trial/Registration/TrialReview.aspx?id=308043>

**Andrews 2011b**

Andrews G. Internet-based treatment of Panic Disorder and/or agoraphobia (the revised Panic program): A randomised controlled study exploring the role of a clinician. <https://www.anzctr.org.au/Trial/Registration/TrialReview.aspx?id=347482>

**Barlow 2010**

Barlow DH. Efficacy evaluation of a unified transdiagnostic treatment for anxiety disorders. <https://clinicaltrials.gov/ct2/show/NCT01243606>

**Botella ONGOING**

Botella C. Efficacy of a Transdiagnostic Internet-based Treatment for Emotional Disorders vs Treatment as Usual in Specialized Care: a Randomized Control Trial. <http://clinicaltrials.gov/show/NCT02345668>

**Carlbring ONGOING**

Lindner P. Guided and Unguided CBT for Social Anxiety Disorder and/or Panic Disorder Via the Internet and a Smartphone Application. <https://clinicaltrials.gov/show/NCT01963806>

**Caspi 2012**

Caspi A. The assessment and treatment of balance impairment using virtual reality (VR) in panic disorder patients. <https://clinicaltrials.gov/ct2/show/NCT01677429>

**Ebenfeld ONGOING/b**

Ebenfeld L. Smartphone-based online-training for panicattacks and agoraphobia. German Clinical Trials Register 2013; DRKS00005223

Ebenfeld L, Kleine Stegemann S, Lehr D, Ebert DD, Jazaieri H, van Ballegooijen W, et al. Efficacy of a hybrid online training for panic symptoms and agoraphobia: Study protocol for a randomized controlled trial. Trials 2014; DRKS00005223

**Gensichen 2012**

Gensichen J, Hiller TS, Breitbart J, Teismann T, Brettschneider C, Schumacher U, et al. Evaluation of a practice team-supported exposure training for patients with panic disorder with or without agoraphobia in primary care - study protocol of a cluster randomised controlled superiority trial [2014]. Trials 2014;15:112.

Gensichen J. Evaluation of a practice team-supported, self-managed exposure training for patients with panic disorder and agoraphobia in primary care [Jena-PARADISE (Patient Activation foR Anxiety DISordErs)]. <http://www.isrctn.com/ISRCTN64669297>

**Gros 2014**

Gros DF. Transdiagnostic Psychotherapy for Veterans With Mood and Anxiety Disorders. <https://clinicaltrials.gov/ct2/show/NCT01947647>

Gros DF. Design challenges in transdiagnostic psychotherapy research: Comparing Transdiagnostic Behavior Therapy (TBT) to existing evidence-based psychotherapy in veterans with affective disorders. Contemporary clinical trials 2015;**43**:114-9

Gros DF. Development and initial evaluation of Transdiagnostic Behavior Therapy (TBT) for veterans with affective disorders. Psychiatry Research 2014;**220**:275-82

**Kok 2012**

Kok RN, van Straten A, Beekman A, Bosmans J, de Neef M, Cuijpers P. Effectiveness and cost-effectiveness of web-based treatment for phobic outpatients on a waiting list for psychotherapy: protocol of a randomized controlled trial. <http://www.trialregister.nl/trialreg/admin/rctview.asp?TC=2233>

Kok Robin N, van Straten A, Beekman A, Cuijpers P. Short-term effectiveness of web-based guided self-help for phobic outpatients: Randomized controlled trial. Journal of medical Internet research 2014;**16**(9):85-100

**Mircea ONGOING**

Ciuca A, Crisan LG. PAXonline: A Randomized Controlled Trial Comparing the Efficacy of an Internet-Based Cognitive Behavior Intervention, delivered with or without assistance from a therapist, to waiting-list in Romanian adults with Panic Disorder. <http://www.anzctr.org.au/ACTRN12614000547640.aspx>

**Ravindran ONGOING**

Ravindran A. Efficacy of Transdiagnostic Internet-based CBT for Early Illness Unipolar Depression and Anxiety. <http://clinicaltrials.gov/show/NCT02266693>

**Rollman 2011**

Rollman B. Online Treatments for Mood and Anxiety Disorders in Primary Care. <https://clinicaltrials.gov/ct2/show/NCT01482806>

**Sandell ONGOING**

Sandell R. Psychotherapy outcome and self-selection effects in panic disorder. <https://clinicaltrials.gov/ct2/show/NCT01606592>

**Schreuders 2008**

Schreuders B. Phobias under control. Internet-based guided self-help for treatment of social, agora-, or specific phobia (s): a pilot trial. <http://www.trialregister.nl/trialreg/admin/rctview.asp?TC=1260>

**Teismann 2012**

Teismann T. Cognitive Behavior Therapy vs Exposure in Vivo in the Treatment of Panic Disorder With Agoraphobia. <https://clinicaltrials.gov/ct2/show/NCT01680237>

**Titov 2012**

Titov N. Is internet-delivered treatment specifically for symptoms of panic disorder as effective as internet-delivered treatment for symptoms of both anxiety and depression, and are self-guided versions of these as effective as therapist-guided versions? <https://www.anzctr.org.au/Trial/Registration/TrialReview.aspx?id=362400>

**References of studies awaiting classification**

**Bressi 2010a**

Bressi C, Ciabatti M, Nocito EP, Catenacci E, Porcellana, M, Invernizzi G et al. A preliminary longitudinal study on the importance of early intervention in panic disorder [conference abstract]. In: Journal of Psychosomatic Research [abstracts from the 19th European Congress of Psychiatry, EPA 2011 Vienna Austria, 12-15 Mar]. 2011:610.

**Foley 2006**

Foley D, Baille A, Renner P. In: 29th Australian Association for Cognitive and Behaviour Therapy Annual Conference. Manly, 18-23 October 2006:43.

**Franklin 1990**

Franklin JA. Behavioural therapy for panic disorder. International Journal of Neuroscience 1990;51:138

**Irgens UNPUBLISHED**

Irgens AC. Thought field therapy and cognitive therapy for agoraphobia. https://clinicaltrials.gov/ct2/show/NCT00932919

**Margraf 1991**

Margraf J, Schneider S. Outcome and active ingredients of cognitive-behavioural treatments for panic disorder. In: 25th Conference of the Association for Advancement of Behavior Therapy. New York, 1991

**Richards 1997**

Richards JB, Bickley N, Rees C, Beros P. An investigation of the mechanisms of change in the cognitive behavioural treatment of panic disorder. Health Perspectives: Research Policy and Practice 1997;1(1):35-44

**Roache 1998**

Roache JD, Oswald LM, Stanley MA, Creason DR, Shah NN. Effects of psychotherapy-induced anxiety reduction on alprazolam self-medication behavior. In: CPDD 1998 Annual Meeting. Scottsdale, 1998:121

**Schmidt 2003**

Schmidt NB, McCreary BT, Trakowski JJ, Santiago HT, Woolaway-Bickel K, Ialongo N. Effects of cognitive behavioral treatment on physical health status in patients with panic disorder. Behavior Therapy 2003;**34**(1):49-63

**Schmidt 2004**

Schmidt NB. Interactive computer treatment for panic disorder. <https://clinicaltrials.gov/ct2/show/NCT00063375>

**Strauss 1997**

Strauss WH, Klieser E. Combination of pharmaco- and psychotherapy in the treatment of panic disorder. European Neuropsychopharmachology 1996;6(Suppl 3):206 (O-24-6)

**Vincelli 2003**

Vincelli F, Anolli L, Bouchard S, Wiederhold BK, Zurloni V, Riva G. Experiential cognitive therapy in the treatment of panic disorders with agoraphobia: a controlled study. Cyberpsychology and Behavior 2003;6(3):321-8

**Vincelli 2004**

Vincelli F, Molinari E, Riva G. CBT Virtual Reality Assisted for the Treatment of Panic Disorders with Agoraphobia: A Controlled Study. In: 35th International Meeting of the Society for Psychotherapy Research. Rome, 16-19 June 2004:35

**References of excluded studies**

**Andersson ONGOING**

Andersson G. Internet-administrated treatment of anxiety symptoms for young adults. <https://clinicaltrials.gov/ct2/show/NCT01402258>

**Barlow 2000**

* Barlow DH, Gorman JM, Shear MK, Woods SW. Cognitive-behavioral therapy, imipramine, or their combination for panic disorder. a randomized controlled trial. JAMA 2000;283(19):2529-36

Barlow DH, Gorman JM, Shear MK, Woods SW. ERRATUM: Cognitive-behavioral therapy, imipramine, or their combination for panic disorder. a randomized controlled trial. JAMA 2000;284(19):2450

Barlow DH, Gorman JM, Shear MK, Woods SW. ERRATUM: Cognitive-behavioral therapy, imipramine, or their combination for panic disorder. a randomized controlled trial. JAMA 2001;284(20):2597

Shear MK, Houck P, Greeno C, Masters S. Emotion-focused psychotherapy for patients with panic disorder. American Journal of Psychiatry 2001;158(12):1993-8

**Bélanger 2006**

Bélanger C, Vaillancourt L, Dulude D, Archambault M, Rochfort J, Pecknold J et al. The differential effect of two behavioral therapies on attentional processes of subjects presenting panic disorder [L'effet différentiel de deux thérapies comportementales sur les processus attentionnels de sujets présentant un trouble panique]. Revue Francophone de Clinique Comportamentale et Cognitive 2008;13(2):11-23

**Benecke ONGOING**

Benecke C. Psychoanalytic therapy (PT) and cognitive-behavioral therapy (CBT) in outpatients with anxiety (panic disorder/agoraphobia) and comorbid personality disorders: a multicenter prospective randomized superiority trial. http://www.isrctn.com/ISRCTN12449681

**Borden 1986**

Clum GA, Watkins PL, Borden JW, Broyles S, Hayes J. A comparison of guided imaginal coping and imaginal exposure in the treatment of panic disorder. Journal of Rational-Emotive & Cognitive-Behavior Therapy 1993;11(4):179

**Elsesser 2002**

Elsesser K, Mosch A, Sartory G. Brief psychological treatment for the relief of panic disorder. Behavioural and Cognitive Psychotherapy 2002;30(4):423-30

**Fava 1997**

Fava GA, Savron G, Zielezny M, Grandi S, Rafanelli C, Conti S. Overcoming resistance to exposure in panic disorder with agoraphobia. Acta Psychiatrica Scandinavica 1997;95(4):306-12

**Febbraro 1997a**

Febbraro GA. An investigation of the differential effectiveness of bibliotherapy and self-regulatory treatments in individuals with panic attacks. Dissertation Abstracts International: Section B: The Sciences and Engineering 1997

**Gaudlitz 2015**

Gaudlitz K, Plag J, Dimeo F, Strohle A. Aerobic exercise training facilitates the effectiveness of cognitive behavioral therapy in panic disorder. Depression and anxiety 2015;**32**(3):221-8

**Gloster 2015**

Gloster AT. Acceptance and commitment therapy (ACT) for treatment-resistant panic disorder with agoraphobia. ISRCTN12042066

Gloster AT, Sonntag R, Hoyer J, Meyer AH, Heinze S, Strohle A, Eifert G, Wittchen HU. Treating treatment-resistant patients with panic disorder and agoraphobia using psychotherapy: A randomized controlled switching trial. Psychotherapy and psychosomatics 2015;**84**:100-9

**Ito 2001**

Ito LM, De Araujo LA, Tess VLC, De Barros-Neto TP, Asbahr FR, Marks I. Self-exposure therapy for panic disorder with agoraphobia. British Journal of Psychiatry 2001;178(4):331-6

**Michelson 1996**

Michelson LK, Marchione KE, Greenwald MT, Testa S, Marchione NJ. A comparative outcome and follow-up investigation of panic disorder with agoraphobia: the relative and combined efficacy of cognitive therapy, relaxation training, and therapist-assisted exposure. Journal of Anxiety Disorders 1996;10(5):297-330

Murphy MT, Michelson LK, Marchione K, Marchione N, Testa S. The role of self-directed in vivo exposure in combination with cognitive therapy, relaxation training, or therapist-assisted exposure in the treatment of panic disorder with agoraphobia. Journal of Anxiety Disorders 1998;12(2):117-38

**Newman 1997**

Newman MG, Kenardy J, Herman S, Taylor CB. Comparison of palmtop-computer-assisted brief cognitive-behavioral treatment to cognitive-behavioral treatment for panic disorder. Journal of Consulting and Clinical Psychology 1997;**65**(1):178-83

**Salkovskis 1991**

Salkovskis PM, Clark DM, Hackmann A. Treatment of panic attacks using cognitive therapy without exposure or breathing retraining. Behaviour Research and Therapy 1991;**29**(2):161-6

**Teusch 1996**

Teusch L, Bohme H, Gastpar M. The benefit of an insight-oriented and experiential approach on panic and agoraphobia symptoms. Results of a controlled comparison of client-centered therapy alone and in combination with behavioral exposure. Psychotherapy & Psychosomatics 1997;66:293-301

**eTable1. Characteristics of the included studies**

| **Study ID** | **Participants**  **(N randomised)** | **Treatment** | **Components** | | | | | | | | | | | | **Remission** | **Response** | **Dropouts** |
| --- | --- | --- | --- | --- | --- | --- | --- | --- | --- | --- | --- | --- | --- | --- | --- | --- | --- |
| **Addis 2004** | 38 | CBT |  | pl | ftf | pe | ps | br |  | ive | ine |  | cr |  | X | X | X |
|  | 42 | SP |  | pl | ftf |  | ps |  |  |  |  |  |  |  |  |  |  |
| **Al Kubaisy 1992** | 13 | BT (self + accompained ive) |  | pl | ftf | pe | ps |  |  | ive |  |  |  |  | X | X | X |
|  | 11 | BT (only self-ive) |  | pl | ftf | pe | ps |  |  | ive |  |  |  |  |  |  |  |
|  | 10 | PT (only relaxation) |  | pl | ftf |  | ps |  | mr |  |  |  |  |  |  |  |  |
| **Barlow 1989** | 16 | CBT (without relaxation) |  | pl | ftf |  | ps | br |  |  | ine |  | cr |  | X | X | X |
|  | 24 | CBT (with relaxation) |  | pl | ftf |  | ps | br | mr |  | ine |  | cr |  |  |  |  |
|  | 15 | PT |  | pl | ftf |  | ps |  | mr |  |  |  |  |  |  |  |  |
|  | 16 | WL | w |  |  |  |  |  |  |  |  |  |  |  |  |  |  |
| **Beck 1987** | 16 | APP |  | pl | ftf |  | ps |  |  |  |  |  |  |  |  |  | X |
|  | 13 | CBT |  | pl | ftf | pe | ps | br | mr | ive | ine |  | cr |  |  |  |  |
| **Beck 1992** | 17 | CT |  | pl | ftf | pe | ps |  |  |  |  |  | cr |  | X |  | X |
|  | 16 | WL | w | pl |  |  | ps |  |  |  |  |  |  |  |  |  |  |
| **Beck 1994** | 22 | CT |  | pl | ftf | pe | ps |  |  |  |  |  | cr |  | X | X | X |
|  | 22 | NT |  |  |  |  | ps |  |  |  |  |  |  |  |  |  |  |
|  | 20 | PT |  | pl | ftf |  | ps | br | mr |  |  |  |  |  |  |  |  |
| **Bell 2012** | 16 | SH-CBT |  | pl |  | pe |  |  |  | ive |  |  | cr |  |  |  | X |
|  | 16 | WL | w |  |  |  |  |  |  |  |  |  |  |  |  |  |  |
| **Berger 2014** | 13 | SH-3W (tailored treatment) |  | pl |  | pe |  |  | mr | ive |  |  | cr | 3w | X |  |  |
|  | 16 | SH-3W (disorder-specific treatment) |  | pl |  | pe |  |  | mr | ive |  |  | cr | 3w |  |  |  |
|  | 15 | WL | w |  |  |  |  |  |  |  |  |  |  |  |  |  |  |
| **Bergstrom 2010** | 53 | SH-CBT |  | pl |  | pe |  |  |  | ive | ine |  | cr |  | X | X | X |
|  | 60 | CBT |  | pl | ftf | pe | ps |  |  | ive | ine |  | cr |  |  |  |  |
| **Botella 2004** | 12 | CBT (with ive) |  | pl | ftf | pe | ps | br |  | ive | ine |  | cr |  | X | X | X |
|  | 12 | CBT (with vre) |  | pl | ftf | pe | ps | br |  |  | ine | vre | cr |  |  |  |  |
|  | 13 | WL | w |  |  |  |  |  |  |  |  |  |  |  |  |  |  |
| **Burke 1997** | 20 | BT |  | pl | ftf | pe | ps |  |  | ive |  |  |  |  | X | X | X |
|  | 19 | CBT |  | pl | ftf | pe | ps |  |  | ive |  |  | cr |  |  |  |  |
| **Carlbring 2001** | 21 | SH-CBT |  | pl |  | pe |  | br |  | ive | ine |  | cr |  | X | X | X |
|  | 20 | WL | w |  |  |  |  |  |  |  |  |  |  |  |  |  |  |
| **Carlbring 2003** | 11 | SH-CBT |  | pl |  | pe |  | br |  | ive | ine |  | cr |  | X | X | X |
|  | 11 | SH-PT |  | pl |  | pe |  |  | mr |  |  |  |  |  |  |  |  |
| **Carlbring 2004** | 24 | CBT |  | pl | ftf | pe | ps | br |  | ive | ine |  | cr |  | X | X | X |
|  | 25 | SH-CBT |  | pl |  | pe |  | br |  | ive | ine |  | cr |  |  |  |  |
| **Carlbring 2006** | 30 | SH-CBT |  | pl |  | pe | ps | br |  | ive | ine |  | cr |  | X | X | X |
|  | 30 | WL | w |  |  |  |  |  |  |  |  |  |  |  |  |  |  |
| **Carter 2003** | 17 | CBT |  | pl | ftf | pe | ps | br |  | ive | ine |  | cr |  | X | X | X |
|  | 15 | WL | w |  |  |  |  |  |  |  |  |  |  |  |  |  |  |
| **Choi 2003** | 20 | CBT (with vre) |  | pl | ftf | pe | ps | br | mr | ive | ine | vre | cr |  | X | X | X |
|  | 20 | CBT (without vre) |  | pl | ftf | pe | ps | br | mr | ive | ine |  | cr |  |  |  |  |
| **Clark 1994 (Original)** | 17 | BT |  | pl | ftf | pe | ps |  | mr | ive |  |  |  |  |  |  | X |
|  | 17 | CBT |  | pl | ftf | pe | ps |  |  | ive | ine |  | cr |  |  |  |  |
|  | 16 | WL | w |  |  |  |  |  |  |  |  |  |  |  |  |  |  |
| **Clark 1994 (Original + Re-randomized)** | 21 | BT |  | pl | ftf | pe | ps |  | mr | ive |  |  |  |  | X | X |  |
|  | 21 | CBT |  | pl | ftf | pe | ps |  |  | ive | ine |  | cr |  |  |  |  |
| **Clark 1999** | 15 | CBT (Full Cognitive Therapy) |  | pl | ftf |  | ps |  |  |  | ine |  | cr |  | X | X | X |
|  | 14 | CBT (Brief Cognitive Therapy) |  | pl | ftf | pe | ps |  |  | ive |  |  | cr |  |  |  |  |
|  | 14 | WL | w |  |  |  |  |  |  |  |  |  |  |  |  |  |  |
| **Cottraux 2009 (Original)** | 29 | BT |  | pl | ftf | pe | ps |  |  | ive |  | vre |  |  |  | X | X |
|  | 31 | CBT |  | pl | ftf | pe | ps |  | mr | ive | ine |  | cr |  |  |  |  |
|  | 32 | WL | w |  |  |  |  |  |  |  |  |  |  |  |  |  |  |
| **Cottraux 2009 (Original + Re-randomized)** | 43 | BT |  | pl | ftf | pe | ps |  |  | ive |  | vre |  |  | X |  |  |
|  | 44 | CBT |  | pl | ftf | pe | ps |  | mr | ive | ine |  | cr |  |  |  |  |
| **Craske 1995** | 16 | CBT |  | pl | ftf | pe | ps | br |  |  | ine |  | cr |  | X | X | X |
|  | 14 | SP |  | pl | ftf | pe | ps |  |  |  |  |  |  |  |  |  |  |
| **Craske 1997** | 27 | CBT (with ine) |  | pl | ftf | pe | ps |  |  | ive | ine |  | cr |  | X | X | X |
|  | 23 | CBT (with br) |  | pl | ftf | pe | ps | br |  | ive |  |  | cr |  |  |  |  |
| **Craske 2003** | 34 | CBT (with ive) |  | pl | ftf | pe | ps | br |  | ive | ine |  | cr |  | X |  | X |
|  | 34 | CBT (without ive) |  | pl | ftf | pe | ps | br |  |  | ine |  | cr |  |  |  |  |
| **Craske 2005a** | 27 | CBT |  | pl | ftf | pe | ps | br | mr |  | ine |  | cr |  | X | X | X |
|  | 16 | WL | w |  |  |  | ps |  |  |  |  |  |  |  |  |  |  |
| **De Beurs 1995** | 27 | BT (ive plus br and ine) |  | pl | ftf | pe | ps | br |  | ive | ine |  |  |  | X | X | X |
|  | 21 | BT (only ive) |  | pl | ftf | pe | ps |  |  | ive |  |  |  |  |  |  |  |
| **De Ruiter 1989** | 17 | BT |  | pl | ftf | pe | ps |  |  | ive |  |  |  |  | X | X | X |
|  | 17 | CBT (with ine only) |  | pl | ftf | pe | ps | br | mr |  | ine |  | cr |  |  |  |  |
|  | 15 | CBT (with ine and ive) |  | pl | ftf | pe | ps | br | mr | ive | ine |  | cr |  |  |  |  |
| **Dow 2000** | 45 | CBT (6 sessions) |  | pl | ftf |  | ps | br |  | ive | ine |  | cr |  | X |  |  |
|  | 50 | CBT (6 sessions + computer assisted) |  | pl | ftf |  | ps | br |  | ive | ine |  | cr |  |  |  |  |
|  | 45 | CBT (12 sessions) |  | pl | ftf |  | ps | br |  | ive | ine |  | cr |  |  |  |  |
|  | 46 | WL | w |  |  |  |  |  |  |  |  |  |  |  |  |  |  |
| **Gloster 2010** | 163 | BT (therapist-guided exposure outside the room) |  | pl | ftf | pe | ps |  |  | ive | ine |  |  |  | X | X | X |
|  | 138 | BT (therapist-guided exposure only inside the room) |  | pl | ftf | pe | ps |  |  | ive | ine |  |  |  |  |  |  |
|  | 68 | WL | w |  |  |  |  |  |  |  |  |  |  |  |  |  |  |
| **Goldstein 2000** | 13 | PT |  | pl | ftf | pe | ps |  | mr |  |  |  |  |  |  |  | X |
|  | 15 | WL | w |  |  |  |  |  |  |  |  |  |  |  |  |  |  |
| **Gould 1993** | 9 | CBT |  | pl | ftf | pe | ps | br | mr | ive | ine |  | cr |  | X | X | X |
|  | 12 | SH-CBT |  | pl |  | pe |  | br | mr | ive | ine |  | cr |  |  |  |  |
|  | 12 | WL | w |  |  |  |  |  |  |  |  |  |  |  |  |  |  |
| **Gould 1995** | 15 | SH-CBT |  | pl |  | pe |  | br | mr | ive |  |  | cr |  | X | X | X |
|  | 15 | WL | w |  |  |  |  |  |  |  |  |  |  |  |  |  |  |
| **Griegel 1995** | 11 | PT (BRT-I: increasing inspiration rate) |  | pl | ftf | pe | ps | br |  |  |  |  |  |  | X | X | X |
|  | 12 | PT (BRT-S: slowing inspiration rate) |  | pl | ftf | pe | ps | br |  |  |  |  |  |  |  |  |  |
|  | 14 | WL | w |  |  |  |  |  |  |  |  |  |  |  |  |  |  |
| **Hecker 2004** | 17 | SH-CBT |  | pl |  | pe |  | br | mr |  | ine |  | cr |  | X |  | X |
|  | 31 | CBT |  | pl | ftf | pe | ps | br | mr |  | ine |  | cr |  |  |  |  |
| **Helbig2009** | 13 | WL (plus SH-PE) | w | pl |  | pe |  |  |  |  |  |  |  |  | X | X | X |
|  | 12 | WL | w |  |  |  |  |  |  |  |  |  |  |  |  |  |  |
| **Hendriks 2010** | 20 | CBT |  | pl | ftf | pe | ps |  | mr | ive | ine |  | cr |  | X | X | X |
|  | 12 | WL | w |  |  |  |  |  |  |  |  |  |  |  |  |  |  |
| **Hoffart 1995** | 26 | BT |  | pl | ftf | pe | ps | br | mr | ive |  |  |  |  | X | X | X |
|  | 26 | CBT |  | pl | ftf | pe | ps |  |  |  | ine |  | cr |  |  |  |  |
| **Ito 1996** | 12 | BT (ive plus ine) |  | pl | ftf | pe | ps | br |  | ive | ine |  |  |  |  |  | X |
|  | 14 | BT (only ive) |  | pl | ftf | pe | ps | br |  | ive |  |  |  |  |  |  |  |
| **Karekla 2004** | 14 | 3rd Wave |  | pl | ftf | pe | ps | br | mr | ive | ine |  | cr | 3w |  |  | X |
|  | 14 | CBT |  | pl | ftf | pe | ps | br | mr | ive | ine |  | cr |  |  |  |  |
| **Kiropoulos 2006** | 40 | CBT |  | pl | ftf | pe | ps | br |  | ive | ine |  | cr |  | X | X | X |
|  | 46 | SH-CBT |  | pl |  | pe | ps | br |  | ive | ine |  | cr |  |  |  |  |
| **Klosko 1988** | 18 | CBT |  | pl | ftf | pe | ps | br | mr |  | ine |  | cr |  | X | X | X |
|  | 16 | WL | w |  |  |  |  |  |  |  |  |  |  |  |  |  |  |
| **Korrelboom 2013** | 70 | CBT |  | pl | ftf | pe | ps |  |  |  | ine |  | cr |  | X | X | X |
|  | 73 | PT |  | pl | ftf | pe | ps |  | mr |  | ine |  |  |  |  |  |  |
| **Lidren 1994** | 12 | CBT |  | pl | ftf | pe | ps | br | mr | ive |  |  | cr |  | X | X | X |
|  | 12 | SH-CBT |  | pl |  | pe |  | br | mr | ive |  |  | cr |  |  |  |  |
|  | 12 | WL | w |  |  |  |  |  |  |  |  |  |  |  |  |  |  |
| **Malbos 2011** | 9 | BT |  | pl | ftf |  | ps |  |  |  |  | vre |  |  | X | X | X |
|  | 8 | CBT |  | pl | ftf | pe | ps |  |  |  |  | vre | cr |  |  |  |  |
| **Meulenbeek 2008** | 50 | CBT |  | pl | ftf | pe | ps |  | mr | ive | ine |  | cr |  | X | X |  |
|  | 50 | WL | w |  |  |  |  |  |  |  |  |  |  |  |  |  |  |
| **Meuret 2008** | 24 | PT |  | pl | ftf | pe | ps | br |  |  |  |  |  |  | X | X | X |
|  | 19 | WL | w |  |  |  |  |  |  |  |  |  |  |  |  |  |  |
| **Meuret 2010** | 24 | BT |  | pl | ftf | pe | ps | br |  | ive |  |  |  |  |  |  | X |
|  | 23 | CBT |  | pl | ftf | pe | ps |  |  | ive |  |  | cr |  |  |  |  |
| **Meyerbroker 2011** | 19 | CBT (with ine plus vre) |  | pl | ftf | pe | ps |  |  |  | ine | vre | cr |  |  |  | X |
|  | 18 | CBT (with ine plus ive) |  | pl | ftf | pe | ps |  |  | ive | ine |  | cr |  |  |  |  |
|  | 18 | WL | w |  |  |  |  |  |  |  |  |  |  |  |  |  |  |
| **Michelson 1985** | 28 | BT (with only ive) |  | pl | ftf | pe | ps |  |  | ive |  |  |  |  | X | X | X |
|  | 29 | BT (with ive plus mr and br) |  | pl | ftf | pe | ps | br | mr | ive |  |  |  |  |  |  |  |
| **Milrod 2015** | 39 | PT |  | pl | ftf | pe | ps |  | mr |  |  |  |  |  |  | X | X |
|  | 81 | CBT |  | pl | ftf | pe | ps | br |  | ive | ine |  | cr |  |  |  |  |
| **Muncy 1991** | 7 | CT (only CT) |  | pl | ftf | pe | ps |  | mr |  |  |  | cr |  |  |  | X |
|  | 8 | CT (CT plus biofeedback) |  | pl | ftf | pe | ps |  | mr |  |  |  | cr |  |  |  |  |
|  | 4 | WL | w |  |  |  |  |  |  |  |  |  |  |  |  |  |  |
| **Nordin 2010** | 20 | SH-CBT |  | pl |  | pe |  | br |  | ive | ine |  | cr |  | X | X | X |
|  | 20 | WL | w |  |  |  |  |  |  |  |  |  |  |  |  |  |  |
| **Ost 1993** | 16 | BT |  | pl | ftf | pe | ps |  |  | ive |  |  |  |  | X | X | X |
|  | 15 | CBT |  | pl | ftf | pe | ps |  |  | ive |  |  | cr |  |  |  |  |
|  | 15 | PT |  | pl | ftf | pe | ps |  | mr |  |  |  |  |  |  |  |  |
| **Ost 1995** | 19 | CBT |  | pl | ftf | pe | ps |  |  |  | ine |  | cr |  | X | X | X |
|  | 19 | PT |  | pl | ftf | pe | ps |  | mr |  |  |  |  |  |  |  |  |
| **Ost 2004** | 25 | BT |  | pl | ftf |  | ps |  |  | ive |  |  |  |  | X | X | X |
|  | 26 | CBT |  | pl | ftf |  | ps |  |  | ive |  |  | cr |  |  |  |  |
|  | 22 | WL | w |  |  |  |  |  |  |  |  |  |  |  |  |  |  |
| **Perez-Ara 2010** | 14 | CBT (only vre) |  | pl | ftf | pe | ps |  |  |  |  | vre | cr |  | X | X |  |
|  | 15 | CBT (vre plus ine) |  | pl | ftf | pe | ps |  |  |  | ine | vre | cr |  |  |  |  |
| **Power 2000** | 37 | CBT (standard therapist contact) |  | pl | ftf | pe | ps |  |  | ive |  |  | cr |  | X | X | X |
|  | 32 | CBT (minimum therapist contact) |  | pl | ftf | pe | ps |  |  | ive |  |  | cr |  |  |  |  |
|  | 35 | SH-CBT |  | pl |  | pe |  |  |  | ive |  |  | cr |  |  |  |  |
| **Rees 1999** | 20 | APP |  | pl | ftf |  | ps |  |  |  |  |  |  |  |  |  | X |
|  | 20 | PE |  | pl | ftf | pe | ps |  |  |  |  |  |  |  |  |  |  |
| **Reinecke 2013** | 14 | CBT |  | pl | ftf | pe | ps |  |  | ive | ine |  | cr |  | X | X | X |
|  | 14 | WL | w |  |  |  |  |  |  |  |  |  |  |  |  |  |  |
| **Richards 2006** | 12 | SH-CBT |  | pl |  | pe | ps | br | mr | ive | ine |  | cr |  | X | X | X |
|  | 11 | SH-CBT |  | pl |  | pe | ps | br | mr | ive | ine |  | cr |  |  |  |  |
|  | 9 | WL | w |  |  | pe | ps |  |  |  |  |  |  |  |  |  |  |
| **Salkovskis 1999** | 9 | BT |  | pl | ftf | pe | ps |  |  | ive |  |  |  |  | X | X | X |
|  | 9 | CBT |  | pl | ftf | pe | ps |  |  | ive |  |  | cr |  |  |  |  |
| **Schmidt 1997a** | 18 | CBT (with br) |  | pl | ftf | pe | ps | br |  | ive | ine |  | cr |  | X | X | X |
|  | 20 | CBT (without br) |  | pl | ftf | pe | ps |  |  | ive | ine |  | cr |  |  |  |  |
|  | 16 | WL | w |  |  |  |  |  |  |  |  |  |  |  |  |  |  |
| **Schmidt 1997b** | 25 | CBT |  | pl | ftf | pe | ps |  |  | ive | ine |  | cr |  | X |  | X |
|  | 12 | WL | w |  |  |  |  |  |  |  |  |  |  |  |  |  |  |
| **Schmidt 2000** | 32 | CBT |  | pl | ftf | pe | ps |  |  | ive | ine |  | cr |  | X |  | X |
|  | 21 | CBT (plus br) |  | pl | ftf | pe | ps | br |  | ive | ine |  | cr |  |  |  |  |
|  | 24 | WL | w |  |  |  |  |  |  |  |  |  |  |  |  |  |  |
| **Scott 1995** | 15 | CBT |  | pl | ftf |  | ps |  |  |  |  |  |  |  |  |  | X |
|  | 6 | WL | w |  |  |  |  |  |  |  |  |  |  |  |  |  |  |
| **Sharp 2004** | 38 | CBT (group sessions) |  | pl | ftf |  | ps |  |  | ive |  |  | cr |  | X | X | X |
|  | 37 | CBT (individual sessions) |  | pl | ftf |  | ps |  |  | ive |  |  | cr |  |  |  |  |
|  | 22 | WL | w |  |  |  |  |  |  |  |  |  |  |  |  |  |  |
| **Shear 1994** | 37 | CBT |  | pl | ftf | pe | ps | br | mr | ive | ine |  | cr |  | X | X | X |
|  | 29 | SP |  | pl | ftf | pe | ps |  |  |  |  |  |  |  |  |  |  |
| **Telch 1993** | 34 | CBT |  | pl | ftf | pe | ps | br |  |  | ine |  | cr |  | X | X | X |
|  | 33 | WL | w |  |  |  |  |  |  |  |  |  |  |  |  |  |  |
| **Tyrer 1988** | 17 | APP |  | pl | ftf |  | ps |  |  |  |  |  |  |  |  |  | X |
|  | 33 | CT |  | pl | ftf |  | ps |  | mr |  |  |  | cr |  |  |  |  |
| **Williams 1996** | 12 | BT |  | pl | ftf | pe | ps |  |  | ive |  |  |  |  | X | X | X |
|  | 13 | CBT |  | pl | ftf | pe | ps |  |  | ive |  |  | cr |  |  |  |  |
|  | 14 | CT |  | pl | ftf | pe | ps |  |  |  |  |  | cr |  |  |  |  |
|  | 9 | WL | w |  |  |  |  |  |  |  |  |  |  |  |  |  |  |
| **Wims 2010** | 32 | SH-CBT |  | pl |  | pe | ps | br |  | ive | ine |  | cr |  | X | X | X |
|  | 27 | WL | w |  |  |  |  |  |  |  |  |  |  |  |  |  |  |
| **Wollburg 2011** | 19 | PT (lower-CO2 breathing training) |  | pl | ftf | pe | ps | br |  |  |  |  |  |  | X | X | X |
|  | 28 | PT (raise-CO2 breathing training) |  | pl | ftf | pe | ps | br |  |  |  |  |  |  |  |  |  |
|  | 27 | WL | w |  |  |  |  |  |  |  |  |  |  |  |  |  |  |
| **Woolaway-Bickel 2008** | 11 | SH-BT |  | pl |  | pe |  |  |  | ive | ine |  |  |  | X | X | X |
|  | 11 | WL | w |  |  |  |  |  |  |  |  |  |  |  |  |  |  |
| **Studies included only in qualitative analyses** | | | | | | | | | | | | | | | | | |
| **Arch 2012** | ? | 3rd Wave |  |  |  |  |  |  |  |  |  |  |  |  |  |  |  |
|  | ? | CBT |  |  |  |  |  |  |  |  |  |  |  |  |  |  |  |
| **Arntz 2002** | ? | CT |  |  |  |  |  |  |  |  |  |  |  |  |  |  |  |
|  | ? | BT |  |  |  |  |  |  |  |  |  |  |  |  |  |  |  |
| **Bouchard 1996** | ? | BT |  |  |  |  |  |  |  |  |  |  |  |  |  |  |  |
|  | ? | CT |  |  |  |  |  |  |  |  |  |  |  |  |  |  |  |
| **Brown 1997** | ? | CBT |  |  |  |  |  |  |  |  |  |  |  |  |  |  |  |
|  | ? | CT |  |  |  |  |  |  |  |  |  |  |  |  |  |  |  |
| **Creager-Berger 2001** | ? | CBT |  |  |  |  |  |  |  |  |  |  |  |  |  |  |  |
|  | ? | PT |  |  |  |  |  |  |  |  |  |  |  |  |  |  |  |
| **Dreessen 1994** | ? | CT |  |  |  |  |  |  |  |  |  |  |  |  |  |  |  |
|  | ? | PT |  |  |  |  |  |  |  |  |  |  |  |  |  |  |  |
| **Emmelkamp 1986** | ? | BT |  |  |  |  |  |  |  |  |  |  |  |  |  |  |  |
|  | ? | CT |  |  |  |  |  |  |  |  |  |  |  |  |  |  |  |
| **Erickson 2003** | ? | CBT |  |  |  |  |  |  |  |  |  |  |  |  |  |  |  |
|  | ? | WL |  |  |  |  |  |  |  |  |  |  |  |  |  |  |  |
| **Hazen 1996** | ? | CBT (use of the manual in a treatment group led by therapists) |  |  |  |  |  |  |  |  |  |  |  |  |  |  |  |
|  | ? | SH-CBT (use of the manual in a self-help treatment group) |  |  |  |  |  |  |  |  |  |  |  |  |  |  |  |
|  | ? | SH-CBT (individual self- administration of the self-help manual) |  |  |  |  |  |  |  |  |  |  |  |  |  |  |  |
|  | ? | WL |  |  |  |  |  |  |  |  |  |  |  |  |  |  |  |
| **Klein 2001** | 11 | SH-CBT | ? | | | | | | | | | | | |  |  |  |
|  | 12 | NT |  |  |  |  |  |  | ? |  |  |  |  |  |  |  |  |
| **Marchione 1987** | ? | CBT |  |  |  |  |  |  |  |  |  |  |  |  |  |  |  |
|  | ? | BT (only ive) |  |  |  |  |  |  |  |  |  |  |  |  |  |  |  |
|  | ? | BT (ive plus mr) |  |  |  |  |  |  |  |  |  |  |  |  |  |  |  |
| **Petterson 1996** | ? | CBT |  |  |  |  |  |  |  |  |  |  |  |  |  |  |  |
|  | ? | NT |  |  |  |  |  |  |  |  |  |  |  |  |  |  |  |
| **Taylor 1982 (a+b)** | ? | PT |  |  |  |  |  |  |  |  |  |  |  |  |  |  |  |
|  | ? | WL |  |  |  |  |  |  |  |  |  |  |  |  |  |  |  |

**eTable2. Characteristics of ongoing studies**

| **Study ID** | **Details (at the time of the search)** |
| --- | --- |
| **Andrews 2011b** | ONGOING: SH-CBT vs WL |
| **Andrews 2011a** | ONGOING: SH-CBT vs WL. Mixed sample: it could be included if RND will be stratified and separate results will be reported. |
| **Barlow 2010** | Only protocol available. Mixed sample. CBT (disorder specific) vs CBT (unified protocol) vs WL. |
| **Botella ONGOING** | ONGOING: Emotion Regulation Transdiagnostic Protoco (SH-CBT? SH-3W?) vs TAU. Mixed sample: it could be included if RND will be stratified and separate results will be reported. |
| **Carlbring ONGOING** | ONGOING: SH-3rd wave (with minimal therapist contact) vs SH-3rd wave (without minimal therapist contact) vs WL |
| **Caspi 2012** | Only protocol. BT (with VR balance challenge) vs BT (without VR balance challenge) vs CBT. |
| **Ebenfeld ONGOING B** | ONGOING: SH-CBT vs WL. |
| **Gensichen 2012** | Only protocol. CBT vs NT. |
| **Gros 2014** | ONGOING: transdiagnostic CBT vs behavioral activation therapy (BAT) |
| **Kok 2012** | SH-CBT (with therapist contact) vs SH-CBT (without therapist contact) |
| **Mircea ONGOING** | ONGOING: SH-CBT vs SH-CBT vs WL |
| **Ravindran ONGOING** | ONGOING: SH-CBT vs WL. Mixed sample: it could be included if RND will be stratified and separate results will be reported. |
| **Rollman 2011** | ONGOING: SH-CBT vs SH-CBT (plus psychological support) vs NT. Mixed sample: it could be included if RND will be stratified and separate results will be reported. |
| **Sandell ONGOING** | Only protocol. Randomised CBT/PD vs chosen CBT/PD vs WL. Completion expected by 2017. |
| **Schreuders 2008** | ONGOING: apparently SH-CBT vs WL. Mixed sample: it could be included if RND will be stratified and separate results will be reported. |
| **Teismann 2012** | ONGOING: CBT vs BT. |
| **Titov 2012** | ONGOING: SH-CBT (with therapist contact) vs SH-CBT (without therapist contact) |

**eTable3. Characteristics of studies awaiting classification**

| **Study ID** | **Details (at the time of the search)** |
| --- | --- |
| **Bressi 2010a** | Only abstract available. |
| **Foley 2006** | Only abstract available. Mixed sample. How many PD patients were there in the full randomized sample? Was randomization stratified by diagnosis? Are separate results for PD patients available? |
| **Franklin 1990** | Only abstract available |
| **Irgens UNPUBLISHED** | COMPLETED BUT UNPUBLISHED. Only protocol available. TFT (thougt field therapy: not included) vs CBT vs WL. The author prefers not to send us the full report in order to avoid subsequent problems in publishing it (personal communication). |
| **Margraf 1991** | Paper not retrievable. |
| **Richards 1997** | Paper not retrievable. |
| **Roache 1998** | Only abstract available. |
| **Schmidt 2003** | CBT vs WL, Included patients partly overlap with those included in Schimdt 2000 and separate results are not reported. |
| **Schmidt 2004** | Only protocol, apparently completed but unpublished, no reply from author |
| **Strauss 1997** | Only abstract available. It is unlcear whether the study is randomized. |
| **Vincelli 2003** | It is unclear whether patients with anxiety disorders others than PDA are included (Title and inclusion criteria are different). In this case, randomization details and separate results for patients with Panic disorder and/or Agoraphobia are not reported. |
| **Vincelli 2004** | Only abstract available. It is uncler whether this stydy represents a continuation of Vincelli 2003 or is a different trial. |

**eTable 4. Characteristics of excluded studies**

| **Study ID** | **Reason for exclusion (PICO)** | **Details** |
| --- | --- | --- |
| **Andersson ONGOING** | Population | ONGOING (personal communication). Only protocol. Mixed sample. More than 20% of patients are under 18 (personal communication). |
| **Barlow 2000** | Comparison | Drug vs Placebo vs CBT vs CBT+Drug vs CBT+Placebo. In the related study exploring EFT vs CBT, not all patients in EFT arm were randomized. |
| **Bélanger 2006** | Study type | Quasi-randomized design (personal communication). |
| **Benecke ONGOING** | Population | ONGOING: All participants must have a comorbid personality disorder |
| **Borden 1986** | Study type | Assignement to WL was not randomised. |
| **Elsesser 2002** | Study type | Quasi-randomized design (personal communication) |
| **Fava 1997** | Population | The study focuses on refractory patients. |
| **Febbraro 1997a** | Population | Mixed sample including patients with panic symptoms but not fulfilling all DSM-IV criteria for Panic disorder: separate results are not reported |
| **Gaudlitz 2015** | Intervention | Treatment: CBT+Endurance training vs CBT+light excersise |
| **Gloster 2015** | Population | The study focuses on refractory patients. |
| **Ito 2001** | Comparison | Since data for the intervention arms include data from re-randomized WL-patients, data for WL arm cannot be extracted (double-count): the three remaining arms would be BT(I) vs BT(E) vs BT(I+E). Comparison not feasible. Randomization probably not respected: "twenty patients left the trial before week 4 and were replaced", "the final sample of 80 patients included 10 of the WL who were re-randomized". Number of patients originally randomized to each arm unclear. Assessment of the original arms (without the re-randomized and the sobstitutes) not reported. |
| **Michelson 1996** | Study type | Replacements aren't evenly distributed and constitute more than 15% of the final sample. |
| **Newman 1997** | Comparison | CBT (12 sessions) vs CBT (4 sessions + palmtop): same components. Comparison not feasible. |
| **Salkovskis 1991** | Study type | Not RCT (randomly selected multiple baseline design) |
| **Teusch 1996** | Study type | Replacements constitute the 24,5% of the final sample (13 patients on 40) |

**Supplement 5: Details of Risk of Bias (RoB) assessments for each domain and for each study**

eFigure 1

At least two out of three review authors (AP, AT, HI) independently assessed the risk of bias of the included studies using a manual adapted from the tool described in the Cochrane Handbook for Systematic Reviews of Interventions^1^.

The majority of studies did not report sufficient details about random sequence generation and allocation concealment. Only 10 studies provided relevant information, and were rated as being at low risk of selection bias.

We rated the risk of bias in outcome assessment in relation to the measure used as short-term remission: only 11 studies could be rated as being at low risk of bias; 17 were at unclear risk and 30 were at high risk.

We rated the risk of incomplete outcome reporting when at least one out of the three outcomes was available. For 6 studies, we could not clearly assess this domain. Regarding the rest of the studies, half of them were considered to be at low risk. The other half was assessed to be at high risk of bias.

The risk of bias due to selective outcome reporting could be assessed in a minority of cases, for which we were able to retrieve the study protocol in order to check whether all outcomes had been reported as planned. In only two cases we found full coherence between pre-planned and reported outcomes (Cottraux 2009; Hendriks 2010).

In almost half of cases we found studies to be at high risk of researcher allegiance bias, a general proxy of various forms of bias that could affect results in favour of one or more study arms towards which authors may have a vested interest. Studies were rated as being at high risk of researcher allegiance bias when: 1) one or more authors in the study were involved in developing the 'investigational' intervention, 2) the authors developed and wrote a manual for the intervention, or 3) the study was funded or organised by a group or a person who had a clear vested interest in the success of a particular intervention.

Studies were rated as being at high risk of therapist allegiance bias when the therapists appeared to have a vested interested in the success of one treatment over another. The assessment of this kind of risk of bias was particularly difficult, because papers seldom report information regarding this domain: for this reason, we rated most studies as being at unclear risk for this possible source of performance bias.

Another possible source of performance bias regarded the quality of the psychological therapy administered to the various participants within study arms. Usable information to assess this domain were provided in more than half of the studies. These mainly appeared to be at low risk of “treatment fidelity bias”. Instead, studies were rated as being at high risk of fidelity bias when 1) active treatment sessions were not supervised/recorded and rated for fidelity or when 2) only a subset of the active treatment arms were monitored for fidelity and this subset was not chosen randomly.

eFigure 1 summarizes the RoB assessments for each item and for each study: green boxes stand for low RoB assessment; yellow boxes stand for unclear RoB assessment; red boxes stand for High RoB assessment; we left the boxes empty when the RoB assessment was not applicable (e.g. in the case of Blinding of outcome assessment for studies which did not report information about short-term remission). eFigure 2 summarizes the RoB across the includes studies.

eFigure 2. Review authors’ judgements about each risk of bias item, for included studies contributing to the primay outcome, presented as percentages. The defintions of and considerations about judgments for the various risk of bias items are presented in *Supplement* 5.

**Cited references**

1. Higgins JPT, Green S (editors). Cochrane Handbook for Systematic Reviews of Interventions Version 5.1.0. The Cochrane Collaboration, 2011.

**Supplement 6: Assessments of inconsistency and additivity assumption for the three outcomes**

**6a. Short-term remission**

ASSESSMENT OF INCONSISTENCY.

*Treatment level:*

Following the loop-specific approach no loop turned out to be significantly inconsistent. We also employed the design-by-treatment interaction model. The global test showed no evidence for inconsistency ($Q$=5.54, 16 d.o.f., p-value 0.992).

*Component level:*

At the component level, we first applied a loop-specific approach and found one possibly inconsistent loop (*pl+ftf+ps+ive* vs *pl+ftf+ps+cr+ive* vs *wl*); however, the analyses that used a design-by-treatment approach to inconsistency gave $Q$=7.36, 16 d.o.f. and p-value 0.97

We therefore concluded that there was no strong evidence of inconsistency in the data.

ASSESSMENT OF ADDITIVITY ASSUMPTION.

We identified five groups of studies that could be used to assess the validity of the additivity assumption. These comprise studies which have different combinations of components but which under this assumption are estimating the same relative treatment effects. They are presented in the following graphs.


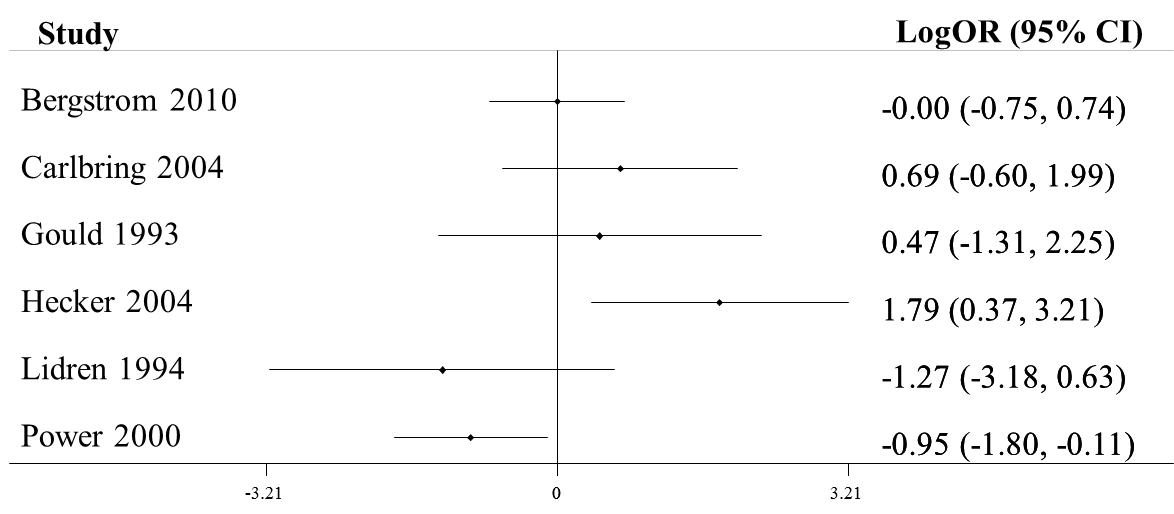


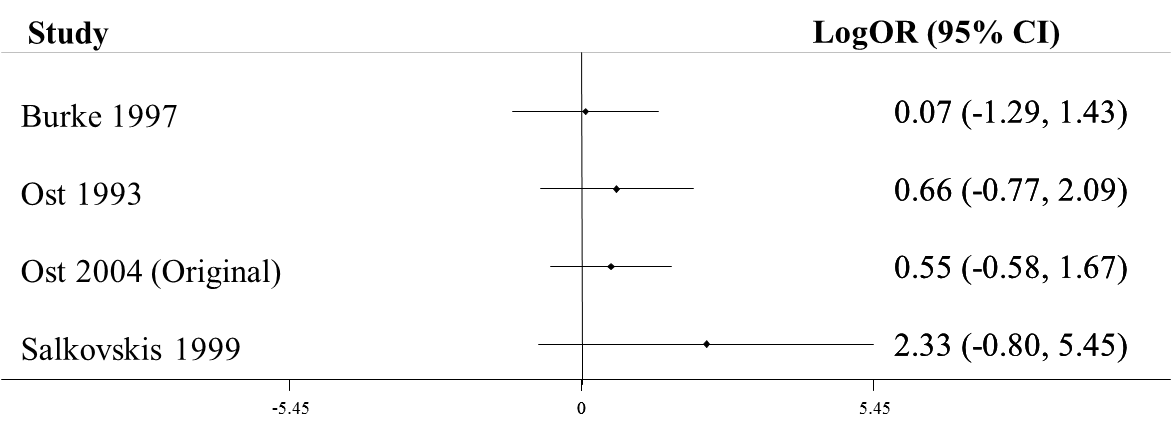


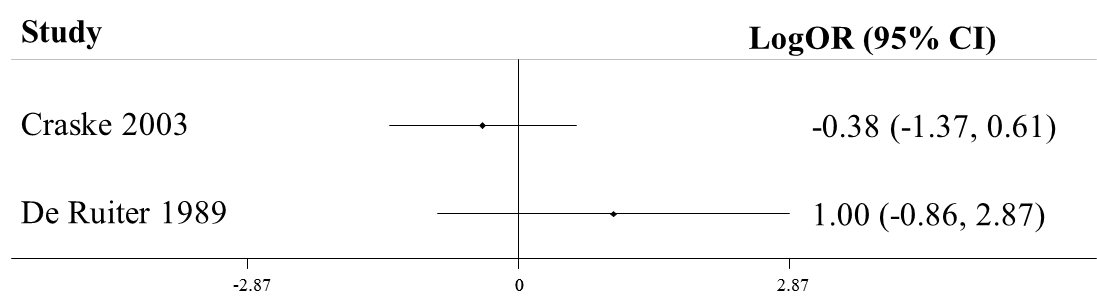


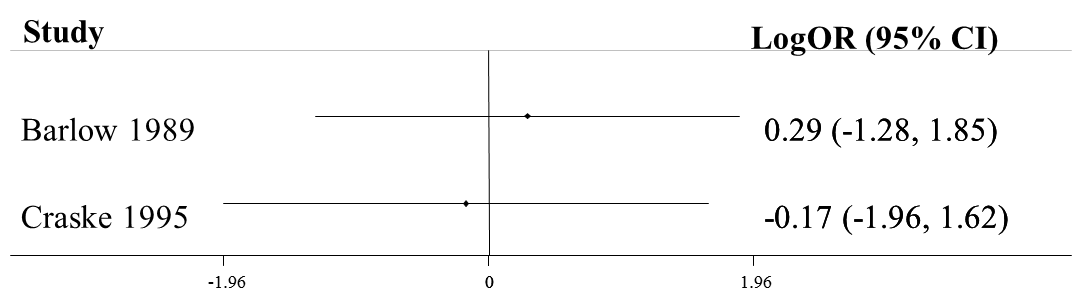


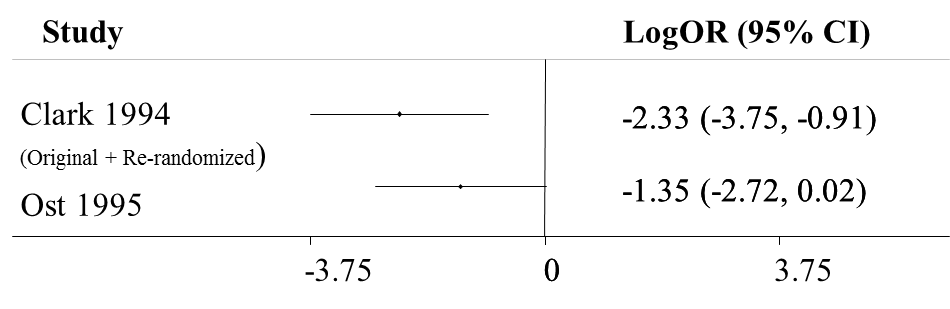


The assumption of additive treatment effects does not seem very plausible for the first set of studies. At the treatment level, these are all CBT vs SH-CBT studies (with different combinations of components), but they provide very different estimates. In the additive treatment effects scenario, all these studies provide evidence for the relative effects of (*ftf*+*ps*+X) vs X, where X is any other combination of other components. Lidren 1994 and Power 2000 show different results than the other 4 studies: they favor CBT while the other 4 studies favor SH-CBT. We did some sensitivity analyses that included two-way interactions between components, trying to find an explanation for this discrepancy. However, the models were less parsimonious than the simple additive model. We therefore concluded that the observed differences in this case are due to usual clinical heterogeneity.

**6b. Short-term response**

ASSESSMENT OF INCONSISTENCY.

*Treatment level:*

We applied a loop-specific approach and no loop turned out to be significantly inconsistent. We also obtained no evidence of inconsistency when applying the design inconsistency model ($Q$=7.95, 15 d.o.f., p-value 0.93).

*Component level:*

At the component level, we first applied the loop-specific approach and found no evidence of inconsistency; the analysis that used the design-by-treatment approach to inconsistency gave $Q$=5.2, 18 d.o.f. and p-value 0.99. We therefore concluded that there was no strong evidence of inconsistency in the data.

ASSESSMENT OF ADDITIVITY ASSUMPTION. Based on the evidence from identified sets of studies with different designs that estimated the same effects (see graphs below), we did not find any strong proof against the additivity assumption.


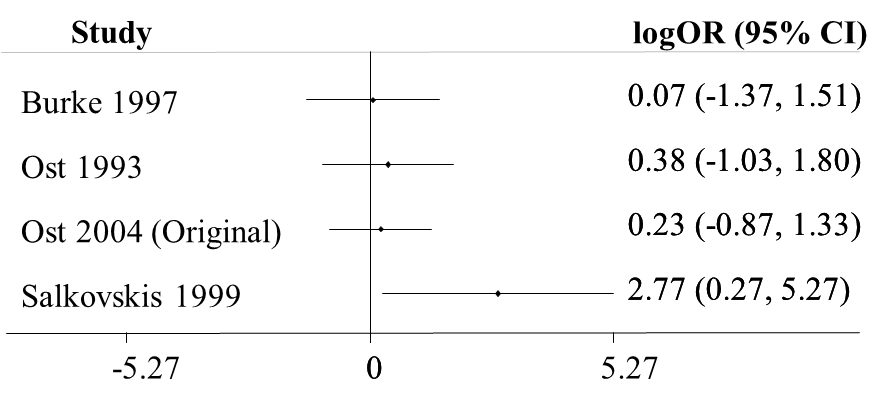


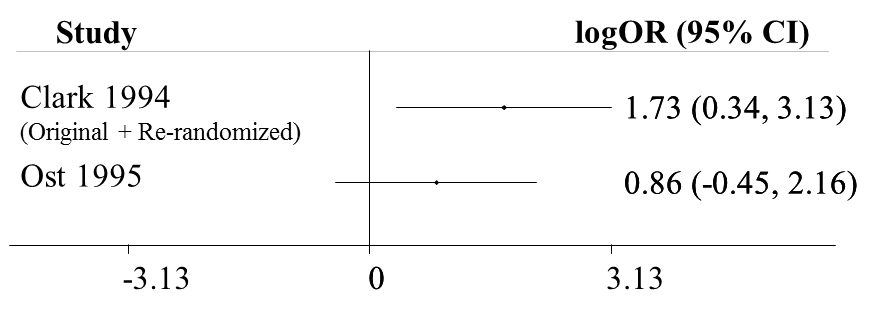


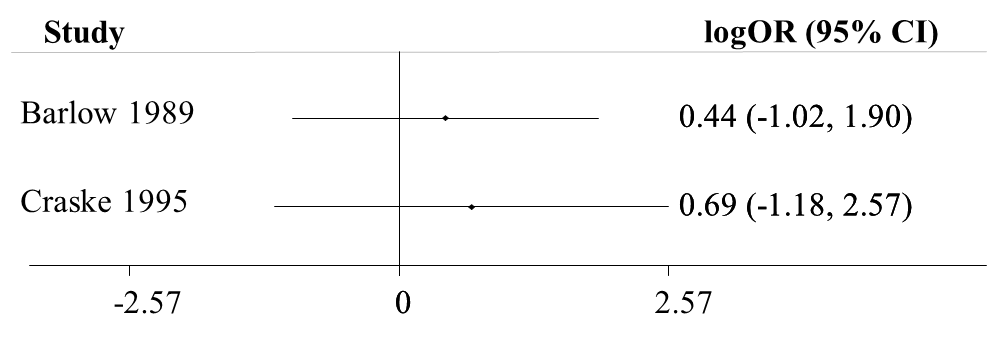


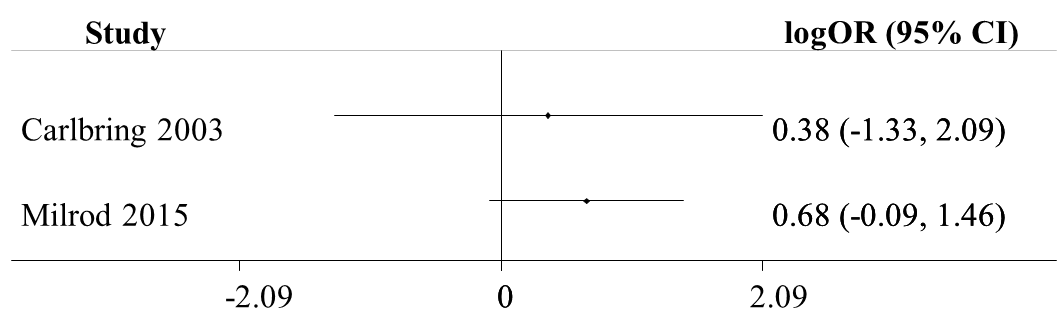


**6c. Short-term dropouts**

ASSESSMENT OF INCONSISTENCY.

*Treatment level:*

We applied a loop-specific approach and no loop turned out to be significantly inconsistent. We also obtained no evidence of inconsistency when applying the design inconsistency model ($Q$=12.666, 17 d.o.f. and p-value 0.758).

*Component level:*

At the component level, we found no evidence of inconsistency when applying the loop-specific approach; the analysis that used the design-by-treatment approach to inconsistency gave $Q$=17.85, 26 d.o.f. and p-value 0.88. We therefore concluded that there was no strong evidence of inconsistency in the data.

ASSESSMENT OF ADDITIVITY ASSUMPTION. Based on the evidence from identified sets of studies with different designs that estimated the same effects (see following graphs), we did not find any strong proof against the additivity assumption.


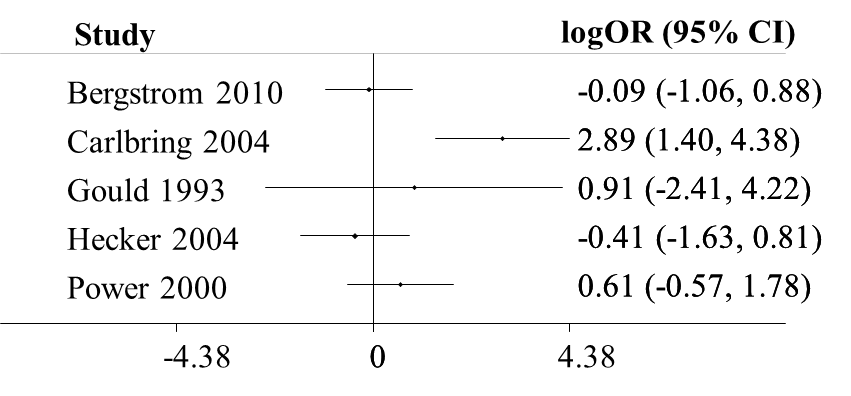


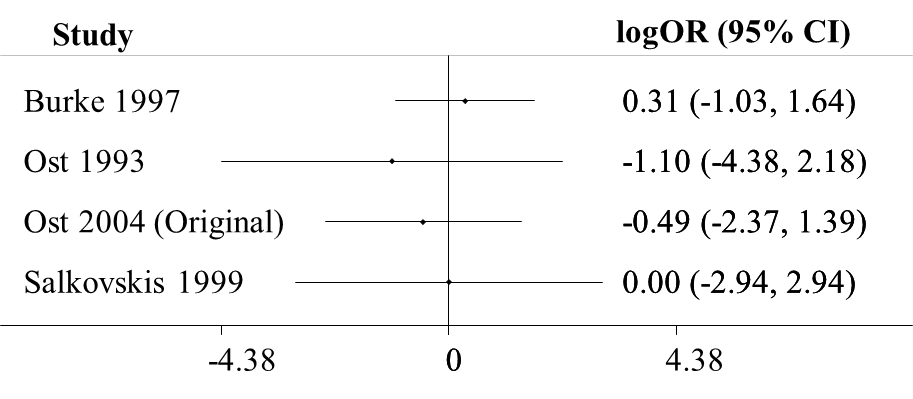


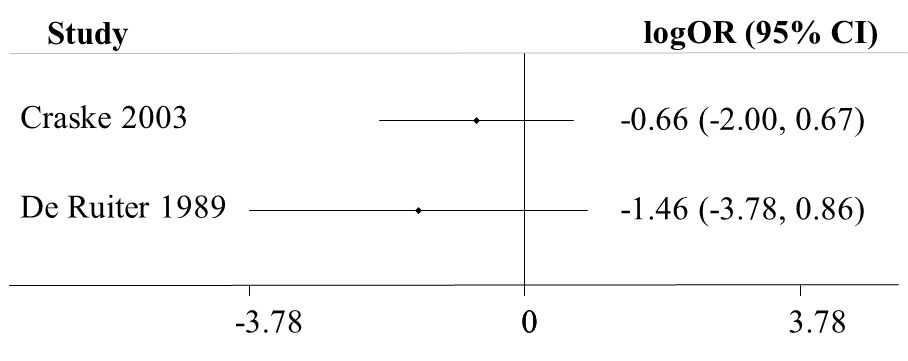


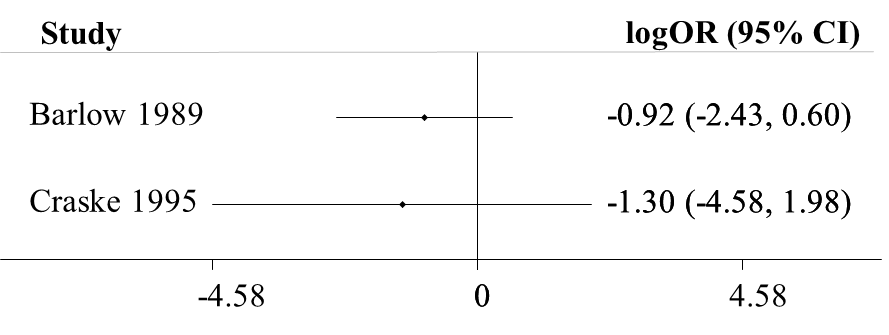


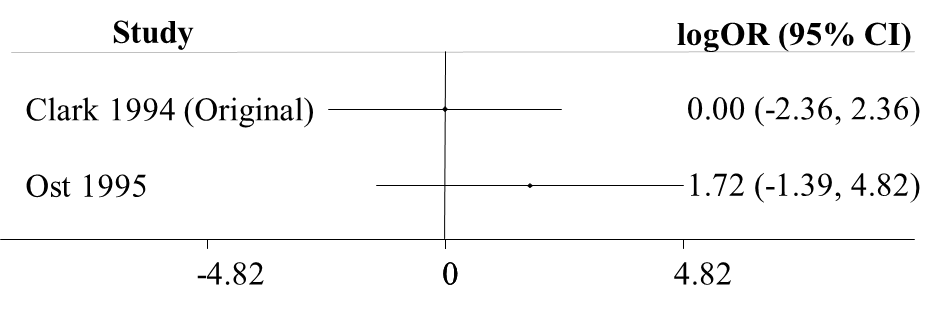


**Supplement 7: Sensitivity analyses examing six clinically suspected interactions**

psychoeducation * interoceptive exposure cognitive restructuring * in vivo exposure

|  | median | lower 95% CI | upper 95% CI |  |  | median | lower 95% CI | upper 95% CI |
| --- | --- | --- | --- | --- | --- | --- | --- | --- |
| *wl* | 0.37 | 0.12 | 1.21 |  | *wl* | 0.32 | 0.10 | 1.05 |
| *3w* | 1.83 | 0.29 | 12.67 |  | *3w* | 2.21 | 0.36 | 13.92 |
| *ftf* | 1.25 | 0.58 | 2.67 |  | *ftf* | 1.39 | 0.64 | 3.13 |
| *pe* | 0.93 | 0.46 | 1.88 |  | *pe* | 0.93 | 0.50 | 1.72 |
| *ps* | 0.80 | 0.34 | 1.83 |  | *ps* | 0.72 | 0.30 | 1.82 |
| *br* | 0.81 | 0.52 | 1.24 |  | *br* | 0.86 | 0.57 | 1.29 |
| *mr* | 0.61 | 0.40 | 0.94 |  | *mr* | 0.52 | 0.34 | 0.81 |
| *ive* | 0.78 | 0.50 | 1.26 |  | *ive* | 0.50 | 0.25 | 1.05 |
| *ine* | 2.19 | 0.73 | 7.88 |  | *ine* | 1.65 | 1.00 | 2.63 |
| *vre* | 0.69 | 0.25 | 2.02 |  | *vre* | 0.80 | 0.28 | 2.34 |
| *cr* | 1.08 | 0.72 | 1.75 |  | *cr* | 0.73 | 0.39 | 1.43 |
| *ps* | 0.91 | 0.32 | 2.44 |  | *ps* | 0.95 | 0.34 | 2.60 |
| *pe*ine* | 0.65 | 0.19 | 1.97 |  | *cr*ive* | 1.96 | 0.83 | 4.45 |

breathing retaining * interoceptive exposure breathing retaining * in vivo exposure

|  | median | lower 95% CI | upper 95% CI |  |  | median | lower 95% CI | upper 95% CI |
| --- | --- | --- | --- | --- | --- | --- | --- | --- |
| *wl* | 0.38 | 0.12 | 1.26 |  | *wl* | 0.37 | 0.10 | 1.19 |
| *3w* | 2.22 | 0.36 | 14.39 |  | *3w* | 1.89 | 0.29 | 13.37 |
| *ftf* | 1.25 | 0.56 | 2.72 |  | *ftf* | 1.25 | 0.56 | 2.78 |
| *pe* | 0.83 | 0.45 | 1.51 |  | *pe* | 0.81 | 0.43 | 1.50 |
| *ps* | 0.79 | 0.33 | 1.93 |  | *ps* | 0.79 | 0.32 | 1.92 |
| *br* | 1.21 | 0.61 | 2.37 |  | *br* | 0.93 | 0.47 | 1.87 |
| *mr* | 0.56 | 0.37 | 0.85 |  | *mr* | 0.60 | 0.40 | 0.91 |
| *ive* | 0.79 | 0.51 | 1.26 |  | *ive* | 0.85 | 0.46 | 1.57 |
| *ine* | 1.87 | 1.05 | 3.27 |  | *ine* | 1.49 | 0.89 | 2.37 |
| *vre* | 0.79 | 0.28 | 2.29 |  | *vre* | 0.69 | 0.24 | 2.08 |
| *cr* | 1.13 | 0.75 | 1.80 |  | *cr* | 1.11 | 0.72 | 1.79 |
| *ps* | 0.88 | 0.32 | 2.44 |  | *ps* | 0.96 | 0.30 | 2.50 |
| *pe*ine* | 0.52 | 0.20 | 1.33 |  | *cr*ive* | 0.85 | 0.37 | 1.93 |

face-to-face * interoceptive exposure face-to-face * cognitive restructuring

|  | median | lower 95% CI | upper 95% CI |  |  | median | lower 95% CI | upper 95% CI |
| --- | --- | --- | --- | --- | --- | --- | --- | --- |
| *wl* | 0.47 | 0.14 | 1.29 |  | *wl* | 0.36 | 0.08 | 1.50 |
| *3w* | 3.11 | 0.49 | 21.29 |  | *3w* | 1.91 | 0.31 | 12.89 |
| *ftf* | 2.09 | 0.85 | 5.95 |  | *ftf* | 1.39 | 0.29 | 5.30 |
| *pe* | 0.82 | 0.45 | 1.48 |  | *pe* | 0.81 | 0.43 | 1.50 |
| *ps* | 0.80 | 0.33 | 1.78 |  | *ps* | 0.78 | 0.29 | 2.07 |
| *br* | 0.81 | 0.53 | 1.21 |  | *br* | 0.83 | 0.54 | 1.27 |
| *mr* | 0.62 | 0.42 | 0.95 |  | *mr* | 0.60 | 0.39 | 0.90 |
| *ive* | 0.77 | 0.50 | 1.19 |  | *ive* | 0.78 | 0.50 | 1.25 |
| *ine* | 3.06 | 1.21 | 8.57 |  | *ine* | 1.50 | 0.92 | 2.43 |
| *vre* | 0.71 | 0.26 | 2.08 |  | *vre* | 0.69 | 0.25 | 2.05 |
| *cr* | 1.09 | 0.72 | 1.68 |  | *cr* | 1.20 | 0.21 | 5.72 |
| *ps* | 0.75 | 0.27 | 2.04 |  | *ps* | 0.92 | 0.25 | 3.32 |
| *pe*ine* | 0.47 | 0.19 | 1.08 |  | *cr*ive* | 0.92 | 0.20 | 5.01 |
